# Supplementary material for: Engineering the Live-Attenuated Polio Vaccine to Prevent Reversion to Virulence
Source: Cell Host Microbe. 2020 May 13;27(5):736–751.e8. doi: 10.1016/j.chom.2020.04.003 (PMC7566161; doi:10.1016/j.chom.2020.04.003)
Supplement: Document S1. Figures S1–S7 [file mmc1.pdf]

Cell Host & Microbe, Volume *27*

Supplemental Information

Engineering the Live-Attenuated Polio Vaccine to Prevent Reversion to Virulence

Ming Te Yeh, Erika Bujaki, Patrick T. Dolan, Matthew Smith, Rahnuma Wahid, John Konz, Amy J. Weiner, Ananda S. Bandyopadhyay, Pierre Van Damme, Ilse De Coster, Hilde Revets, Andrew Macadam, and Raul Andino

Engineering the Live-Attenuated Polio Vaccine to Improve Safety by Preventing Reversion to Virulence

Yeh et al.

Supplementary figures

Figure S1

A

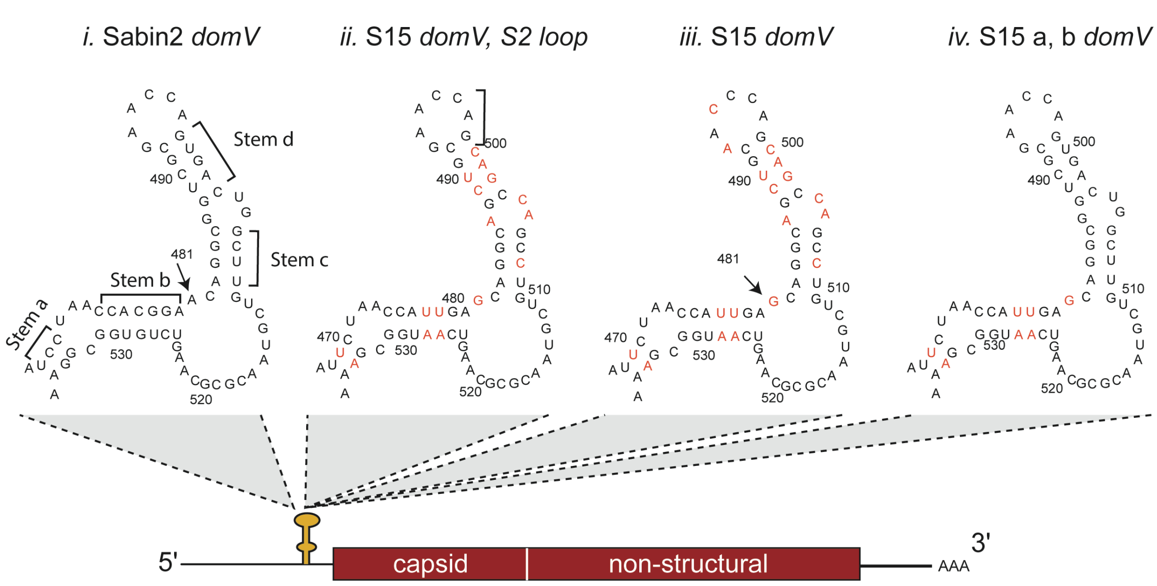

B

Temperature sensitivity

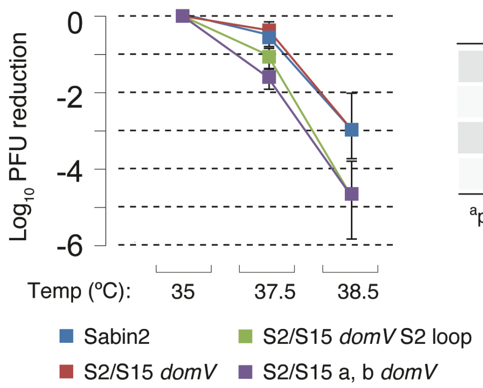

C

Neurovirulence

	Tg66-CBA, i.s.
	PD$_{50}$ (log$_{10}$CCID$_{50}$)
Sabin2	5.9
S2/S15 a, b *domV*	> 7.0 (0/8[a])
S2/S15 *domV* S2 loop	> 7.0 (0/8[a])
S2/S15 *domV*	6.0

[a]paralyzed/total at the highest dose

Figure S1. Related to Figure 1. Modifications within *domV*.

(A) Sabin2 domain V (*domV*) predicted secondary structures and optimized S15. The 5′-UTR *domV* is an RNA stem-loop structure involving nucleotides 468–535. Nucleotide

481A, the major attenuating mutation in Sabin2, is identified by arrow. Nucleotide differences between Sabin2 and modified *domV* are shown in red. *DomV* of Sabin2 was engineered to have all but the top-loop (S15 *domV*, S2 loop, *ii*), the entire domain (S15 *domV*, *iii*), or stems a and b and connecting loops (S15a, b *domV*, *iv*) replaced by the corresponding region from S15 (1).

(**B, C**) Temperature sensitivity (B) and neurovirulence (C) of the mutant viruses were evaluated in Vero cells and by intraspinal (i.s.) inoculation of poliovirus into susceptible Tg66 mice, respectively (STAR methods).

Figure S2

A

	Cre1	Cre1a	Cre2	Cre3	Cre4	Cre5
ΔΔG:	0.0	-0.4	-2.8	-4.7	-6.9	-10.0

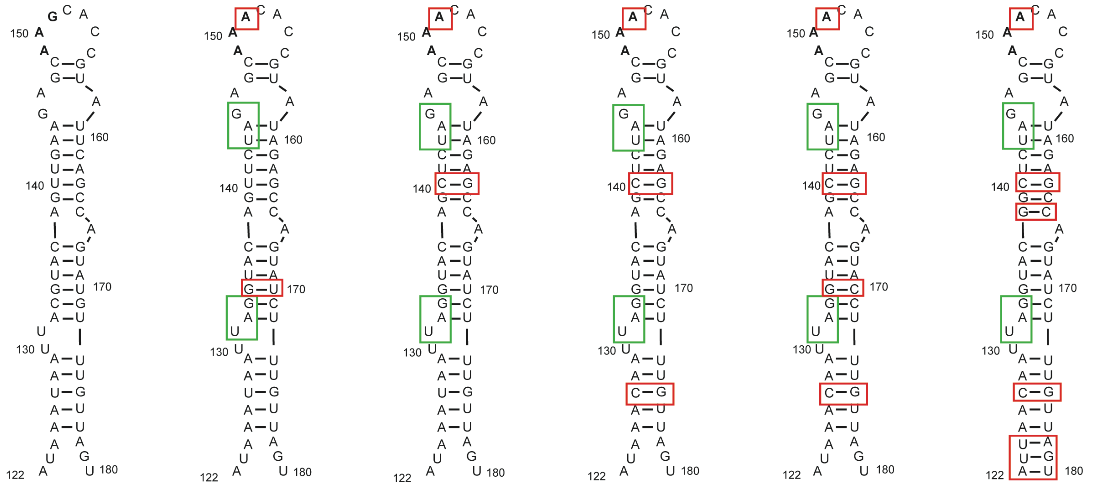

B

Temperature sensitivity

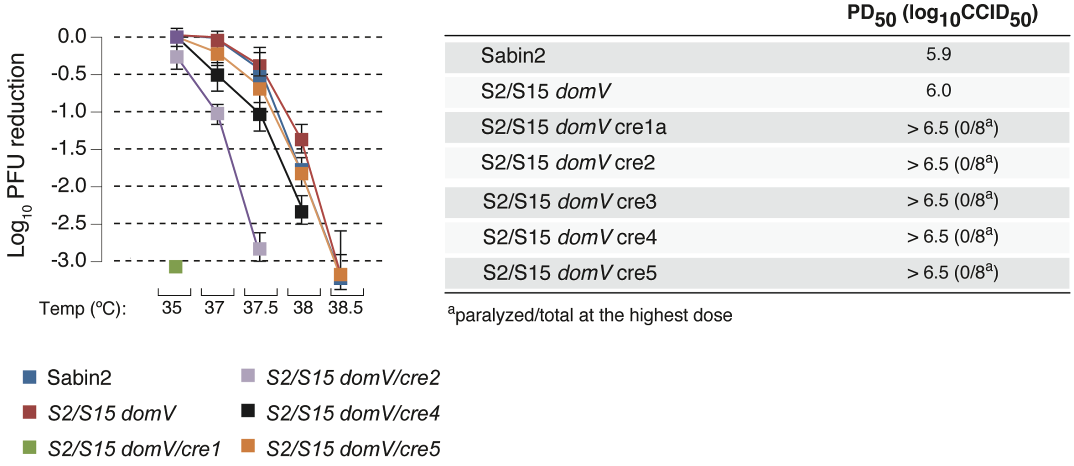

C

Neurovirulence

	Tg66-CBA, i.s.
	PD_{50} ($log_{10}CCID_{50}$)
Sabin2	5.9
S2/S15 *domV*	6.0
S2/S15 *domV* cre1a	> 6.5 (0/8[a])
S2/S15 *domV* cre2	> 6.5 (0/8[a])
S2/S15 *domV* cre3	> 6.5 (0/8[a])
S2/S15 *domV* cre4	> 6.5 (0/8[a])
S2/S15 *domV* cre5	> 6.5 (0/8[a])

[a]paralyzed/total at the highest dose

Figure S2. Related to Figure 1 and 5. CRE Optimization.

(**A**) RNA secondary structures of *cre* and serially optimized *cre* 1–5 with optimized base-pairs shown in red rectangles and "in-frame" STOP codons in green rectangles.

(**B, C**) Temperature sensitivity in Vero cells (B) and neurovirulence in Tg66 mice (C) of Sabin2 with *cre* mutants were compared to select sufficiently fit *cre5* for further analysis.

Figure S3

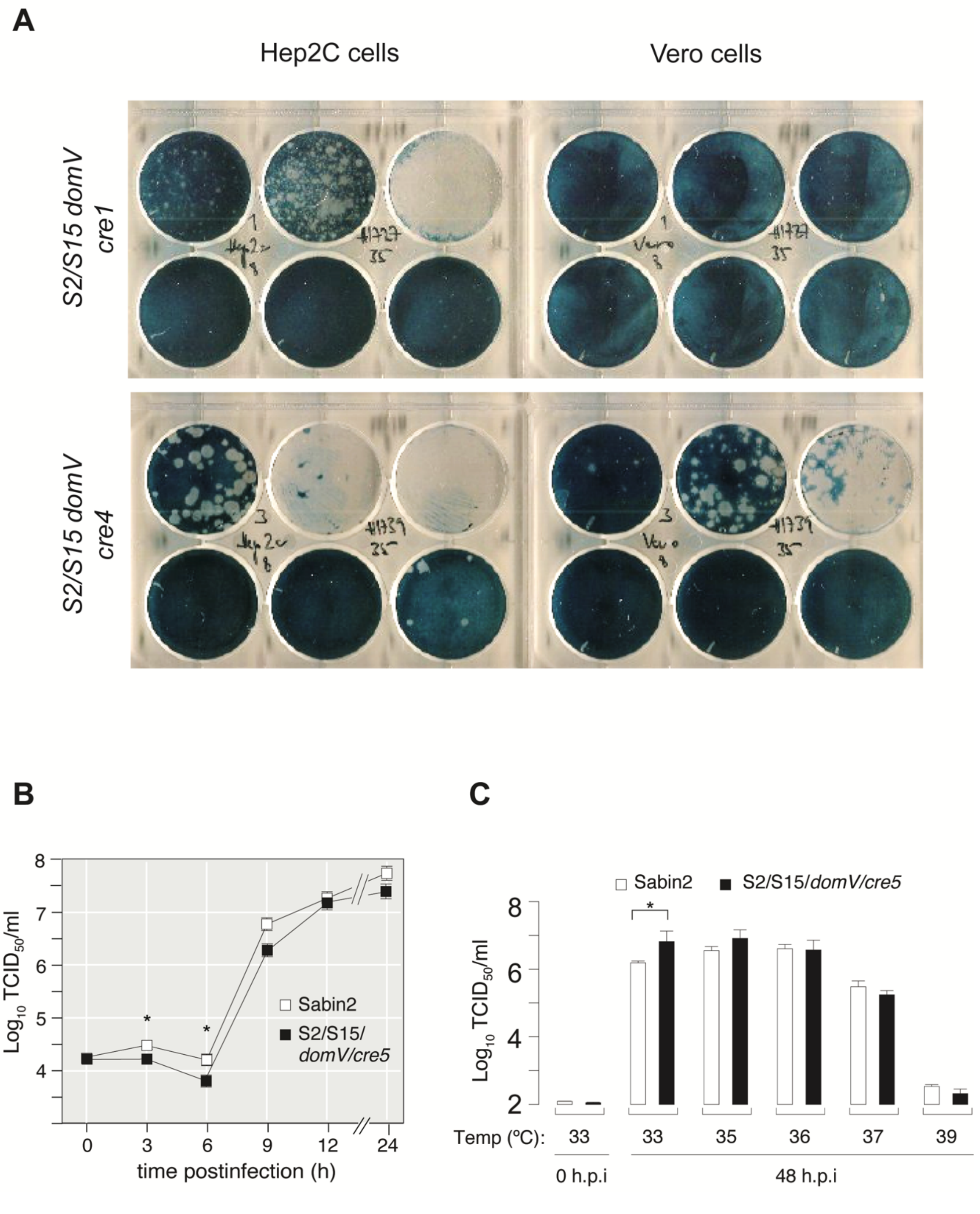

Figure S3. Related to Figure 1 and 5. Plaque Morphology and Replication Phenotype of *cre* Variants.

(**A**) Plaque morphology of S2/S15 *domV/cre1* and *cre4*. For each virus equivalent, 10-fold dilutions were plated from top-right to bottom-left in the 6-well plates of Hep2c and Vero cells. (**B**) Vero cells were infected with S2/S15 *domV/cre5* and Sabin2 viruses at m.o.i. of 10 at 33 ºC for one-step growth analysis. Asterisks indicate significant difference as compared to Sabin2; *p* values are 0.8796, 0.0238, 0.0136, 0.3419, 0.8796, and 0.5938 for 0, 3, 6, 9, 12, and 24-h post-infection, respectively.

(**C**) Vero cells were infected with S2/S15 *domV/cre5* and Sabin2 viruses at an m.o.i. of 0.01 under different temperatures and harvested 48 h after infection to compare virus replication yields. Asterisk represents significant difference as compared to Sabin2; *p* = 0.0359, 0.3061, 0.6433, 0.3165, and 0.4295 for 33, 35, 36, 37 and 39°C, respectively.

Figure S4

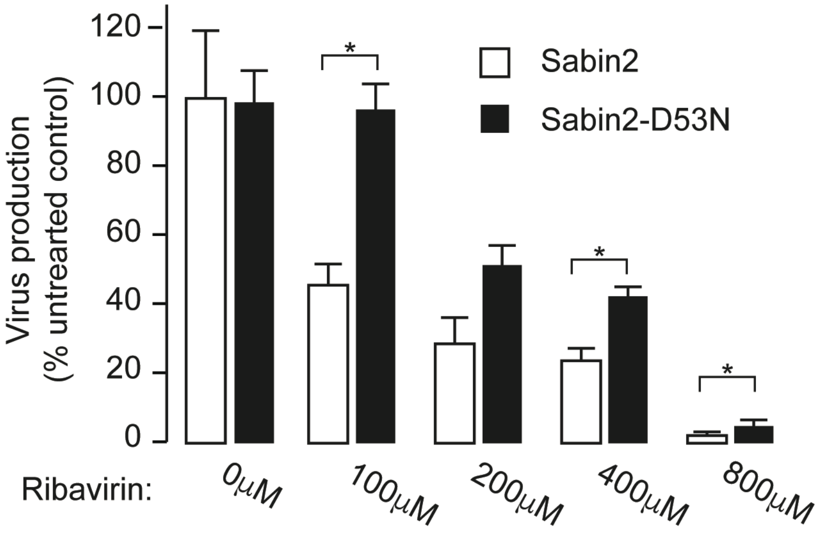

Figure S4. Related to Figure 2. Ribavirin Resistant Mutants.

To test for replication fidelity, we used a ribavirin-sensitivity test. Among the 11 fidelity candidates isolated, Sabin2-D53N showed the most significant increase in resistance to mutagen (ribavirin) of the tested concentrations. Asterisks indicate significant difference as compared to Sabin2; p = 0.9990, 0.0267, 0.1237, 0.0054, and 0.0065 for 0, 100, 200, 400, and 800 μM, respectively.

Figure S5

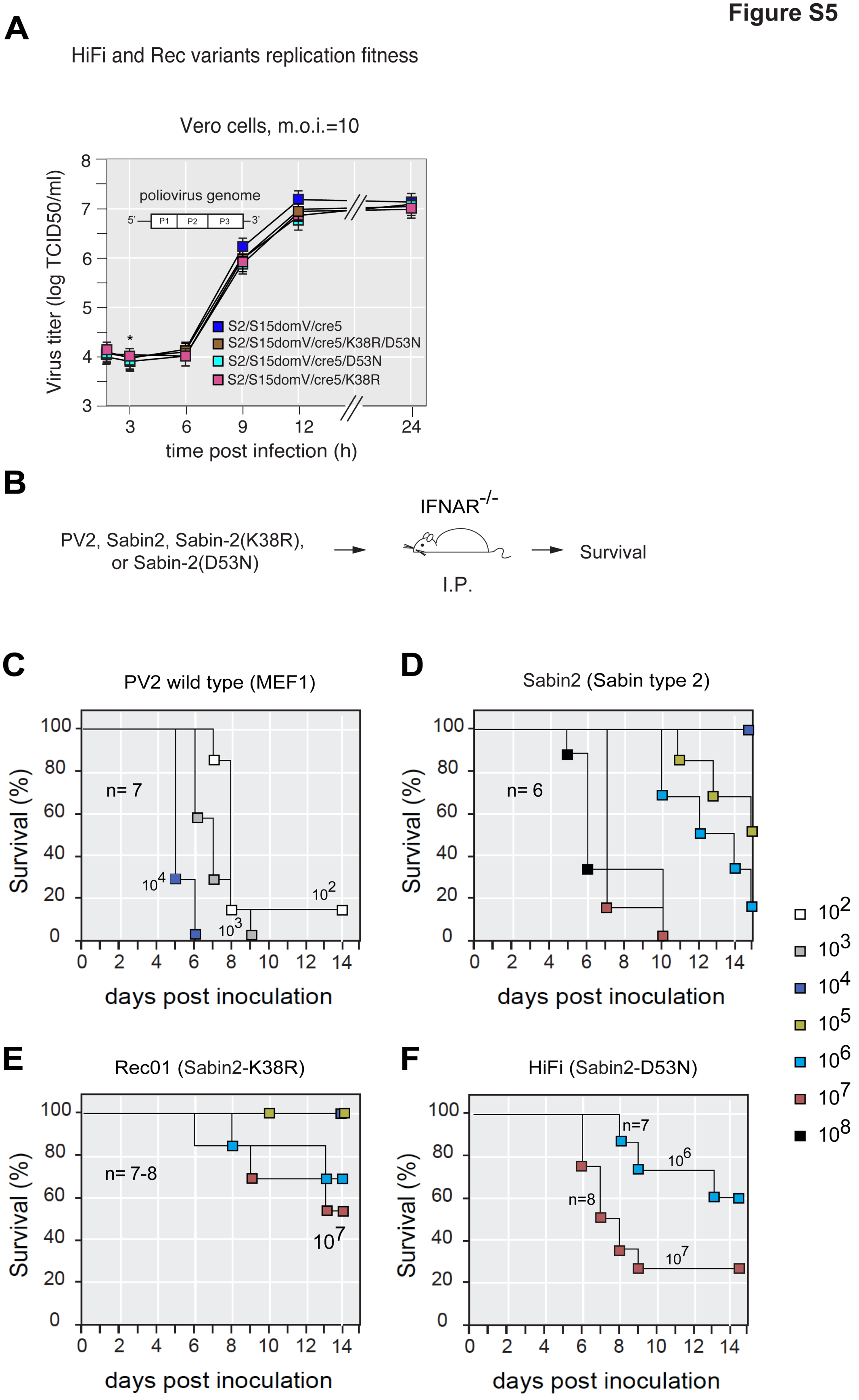
A
HiFi and Rec variants replication fitness
Vero cells, m.o.i.=10
poliovirus genome
5' P1 P2 P3 3'
S2/S15domV/cre5
S2/S15domV/cre5/K38R/D53N
S2/S15domV/cre5/D53N
S2/S15domV/cre5/K38R
Virus titer (log TCID50/ml)
time post infection (h)
B
PV2, Sabin2, Sabin-2(K38R), or Sabin-2(D53N)
IFNAR-/-
I.P.
Survival
C
PV2 wild type (MEF1)
n= 7
D
Sabin2 (Sabin type 2)
n= 6
E
Rec01 (Sabin2-K38R)
n= 7-8
F
HiFi (Sabin2-D53N)
n=7
n=8
Survival (%)
days post inoculation
10^2
10^3
10^4
10^5
10^6
10^7
10^8

Figure S5. Related to Figure 2. Replication and Virulence of *HiFi* and *Rec1* Viruses.

(**A**) Effects of D53N and K38R mutation on Sabin2 replication were determined by one-step growth analysis by comparing replication kinetics. Vero cells were infected at an m.o.i. of 10 at 33 ºC. Line plots show means ± standard deviation (SD) of triplicates. * $p = 0.0017$ for difference between S2/S15doV/cre5 and S2/S15doV/cre5-K38R at 3-h post infection.

(**B**) Virulence of PV2, Sabin2 and Sabin2 mutants were compared in an IFNR-KO mouse model.

(**C-F**) Survival of mice intraperitoneally (i.p.) inoculated with PV2 wild type (C), Sabin2 (D), Sabin2-*Rec1* (E) and Sabin2-*HiFi3* (F) were monitored for 14 days. Log-rank test was performed to compare the virulence of Sabin2 and mutants. *p* values were 0.0076 for Rec1 and Sabin2 at doses of 10^7, 0.16 at 10^6, and 0.0387 at 10^5 (E). *p* value = 0.7757 for HiFi3 at doses of 10^7 and 0.3480 at 10^6 (F).

Figure S6

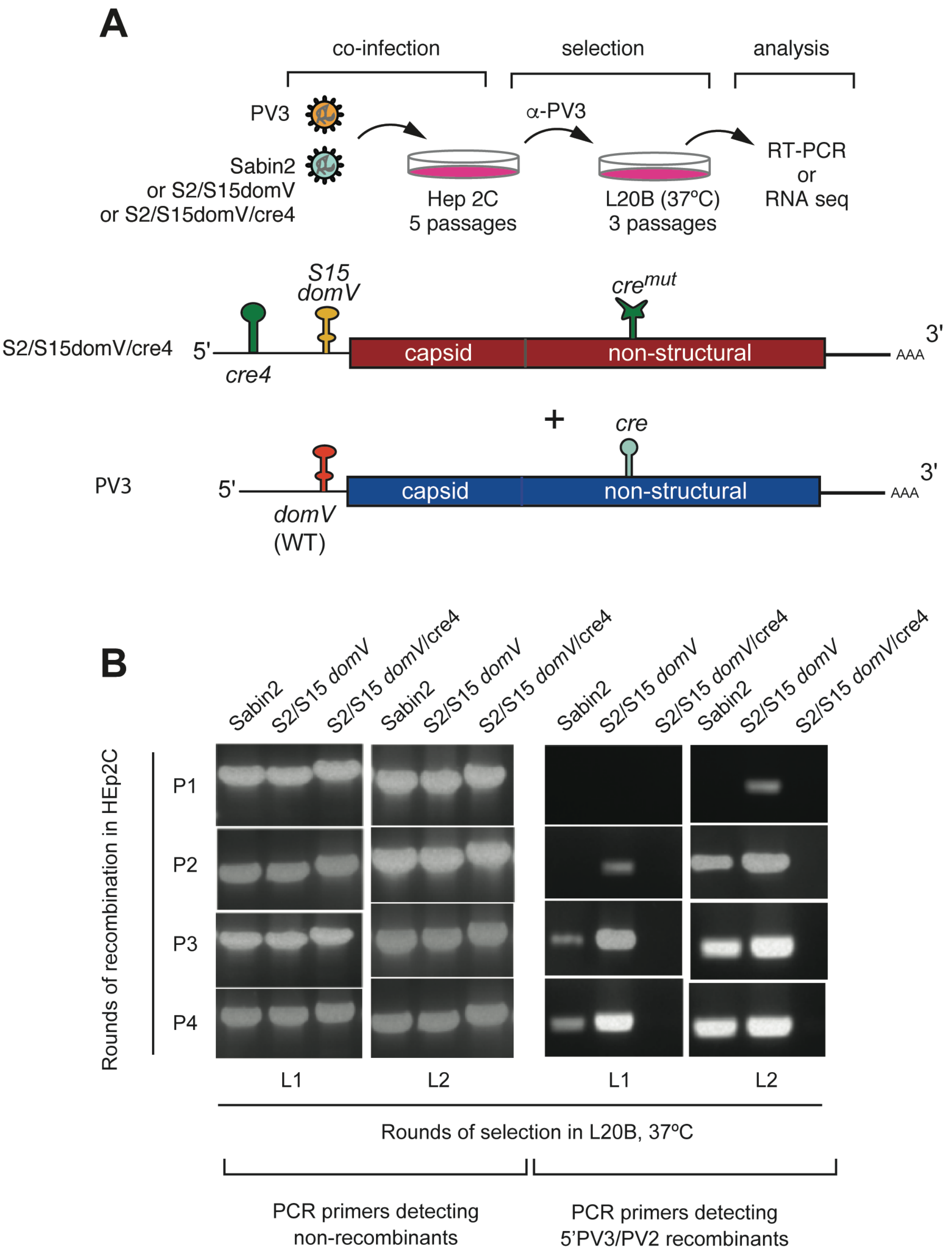

Figure S6. Related to Figure 5. Recombination Assay.

(**A**) Diagram of the experimental procedure to test the effects of CRE relocation on recombination. HEp2C cells were coinfected with PV3 plus one of the type 2 viruses at 33 ºC. Viral progeny was neutralized with anti-PV3 antibodies and further selected in L20B cells at 37 ºC after each HEp2c passage. The resulting virus populations were analyzed by RT-PCR and deep sequencing.

(**B**) Recombination between Sabin2 and PV3 was analyzed by RT-PCR after each passage in Hep2C (P1-P4) and after selection in L20B cells (L1-L2) or full-length NGS. Primer sets specific to non-recombinants (left panel), and 5′PV3/PV2 recombinants (right panel) were used in RT-PCR analysis.

Figure S7

	composition of domV	PD50 (log $CCID_{50}$)
PV3 + Sabin2	40% PV3; 60% Sabin-2	1.8
PV3 + S2/S15domV	70% PV3; 30% S2/S15domV	1.8
PV3 + S2/S15domV/cre5	100% S2/S15domV/cre5	5.6

Figure S7. Related to Figure 5. Characterization of Progeny Virus from Recombination Assay.

Sequence composition of *domV* in virus populations obtained after five recombination passages in HEp2c cells and two selection passages in L20B cells (H5L2) were determined by deep sequencing. Percentage of *domV* sequence mapped to PV3, Sabin2, S2/S15*domV*, or S2/S15*domV/cre5* is shown. Levels of neurovirulence of the obtained virus populations are compared between conditions using PD_{50} determined with the Tg66 mouse model.
