# Supplementary material for: Engineering the Live-Attenuated Polio Vaccine to Prevent Reversion to Virulence
Source: Cell Host Microbe. 2020 May 13;27(5):736–751.e8. doi: 10.1016/j.chom.2020.04.003 (PMC7566161; doi:10.1016/j.chom.2020.04.003)
Supplement: Document S2. Article plus Supplemental Information [file mmc2.pdf]

Article

Cell Host & Microbe

Engineering the Live-Attenuated Polio Vaccine to Prevent Reversion to Virulence

Graphical Abstract

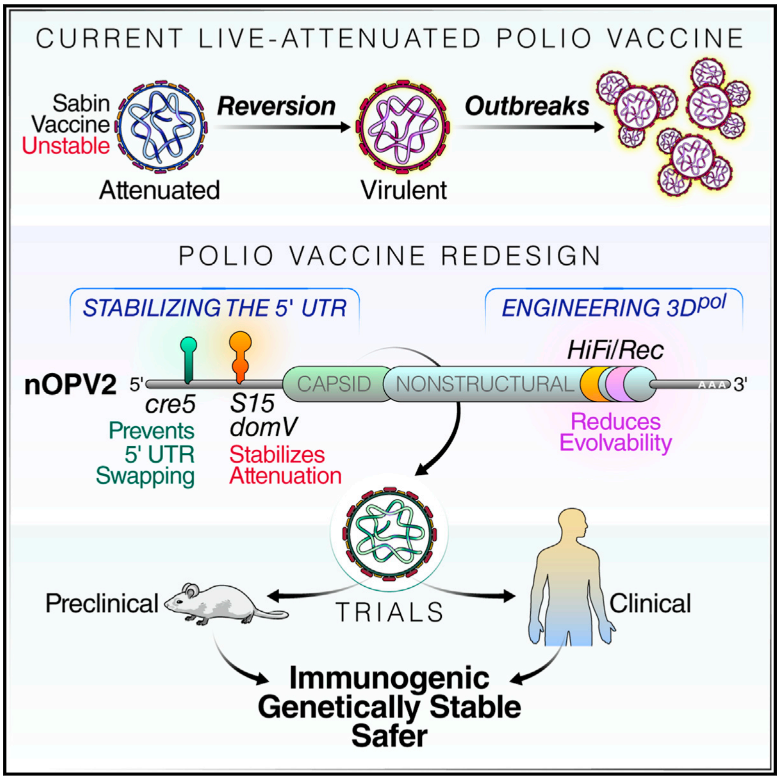

Highlights

- The Sabin vaccine protects from poliomyelitis but can regain neurovirulence
- Specific modifications into the Sabin2 genome increase its genetic stability
- Slowing down Sabin2 virus evolution prevents reversion and leads to a safer vaccine
- The new strain (nOPV2) is safe and immunogenic in preclinical and clinical studies

Authors

Ming Te Yeh, Erika Bujaki, Patrick T. Dolan, ..., Hilde Revets, Andrew Macadam, Raul Andino

Correspondence

andrew.macadam@nibsc.org (A.M.), raul.andino@ucsf.edu (R.A.)

In Brief

The live-attenuated oral poliovirus vaccine (OPV; Sabin vaccine) can revert to neurovirulent variants, causing safety concerns. Yeh et al. engineer a poliovirus vaccine strain (nOPV2) that preserves the antigenic and immunogenic characteristics of Sabin2 while stabilizing determinants of attenuation and reducing evolvability. nOPV2 is safe and immunogenic in preclinical studies.

Yeh et al., 2020, Cell Host & Microbe *27*, 736–751
May 13, 2020 © 2020 Published by Elsevier Inc.
https://doi.org/10.1016/j.chom.2020.04.003

CellPress

CellPress

Cell Host & Microbe

Article

Engineering the Live-Attenuated Polio Vaccine to Prevent Reversion to Virulence

Ming Te Yeh,[1] Erika Bujaki,[2] Patrick T. Dolan,[1] Matthew Smith,[2] Rahnuma Wahid,[3] John Konz,[3] Amy J. Weiner,[4] Ananda S. Bandyopadhyay,[4] Pierre Van Damme,[5] Ilse De Coster,[5] Hilde Revets,[5] Andrew Macadam,[2,*] and Raul Andino[1,6,*]
[1]Department of Microbiology and Immunology, University of California, San Francisco, San Francisco, CA 94158, USA
[2]National Institute for Biological Standards and Control (NIBSC), South Mimms, Herts EN6 3QG, UK
[3]Center for Vaccine Innovation and Access, PATH, Seattle, WA 98121, USA
[4]Bill and Melinda Gates Foundation, Seattle, WA 98109, USA
[5]Centre for the Evaluation of Vaccination, Vaccine and Infectious Disease Institute, University of Antwerp, Antwerp 2610, Belgium
[6]Lead Contact
*Correspondence: andrew.macadam@nibsc.org (A.M.), raul.andino@ucsf.edu (R.A.)
https://doi.org/10.1016/j.chom.2020.04.003

SUMMARY

The live-attenuated oral poliovirus vaccine (OPV or Sabin vaccine) replicates in gut-associated tissues, eliciting mucosa and systemic immunity. OPV protects from disease and limits poliovirus spread. Accordingly, vaccination with OPV is the primary strategy used to end the circulation of all polioviruses. However, the ability of OPV to regain replication fitness and establish new epidemics represents a significant risk of polio reemergence should immunization cease. Here, we report the development of a poliovirus type 2 vaccine strain (nOPV2) that is genetically more stable and less likely to regain virulence than the original Sabin2 strain. We introduced modifications within at the 5′ untranslated region of the Sabin2 genome to stabilize attenuation determinants, 2C coding region to prevent recombination, and 3D polymerase to limit viral adaptability. Prior work established that nOPV2 is immunogenic in preclinical and clinical studies, and thus may enable complete poliovirus eradication.

INTRODUCTION

Live-attenuated vaccines are highly effective in affording protection against disease and in stopping the epidemic spread of pathogenic viruses. However, developing safe and effective live-attenuated vaccines has been a challenging trial-and-error process. One of the most successful examples, Sabin's antipolio vaccine, has been used to nearly eradicate poliomyelitis. In 1988, an estimated 350,000 persons were paralyzed by wild polioviruses (WPVs). By 2019, the global incidence has been reduced by more than 99%, to only a few cases of WPV-related paralysis each year (112 cases in 2019) (Polio Global Eradication Initiative, 2020). Global eradication of poliomyelitis is nearly within reach. The remaining cases occur in endemic countries and countries re-infected via importations. Failure to eradicate polio in the last remaining endemic hotspots could result in resurgence of the disease, with poliovirus spreading to cause outbreaks in polio-free countries around the world.

Three serotypes exist for poliovirus. Wild-type (WT) 2 (WPV2) and WT 3 (WPV3) are considered eradicated, but WPV1 still circulates, causing disease in Afghanistan, Pakistan, and Nigeria (Polio Global Eradication Initiative, 2020). Two vaccines, live-attenuated oral poliovirus vaccine (OPV) and inactivated poliovirus vaccine (IPV), are used to protect against polio. In most countries, a combination of bivalent OPV (type 1 and type 3) and IPV is used. OPV is cheaper than IPV. It replicates in the recipient's gut, eliciting superior primary intestinal immunity, compared with IPV, and thus is more effective to prevent transmission of wild viruses. OPV confers contact immunity through indirect immunization of unvaccinated persons from viruses shed by vaccinees (Burns et al., 2014; Guest et al., 2004; Lauring et al., 2010), and it is administered in oral drops, which makes it easier to administer, store, and transport than IPV injections. Thus, OPV has been the central strategy used to end the circulation of all polioviruses.

Although OPV has been very effective in bringing WPVs to the brink of eradication (Minor, 2012), the global eradication program might be at risk because OPV can evolve and regain transmissibility and virulence. In regions with low vaccine coverage, poliomyelitis outbreaks associated with circulating vaccine-derived polioviruses (cVDPVs) have been reported over the past two decades (Kew et al., 2002, 2005; Stern et al., 2017). In 2019, more than 300 cases of paralytic poliomyelitis were reported (Polio Global Eradication Initiative, 2020). Most of these incidents were caused by variants of Sabin type 2 (Sabin2), which rapidly spreads in settings of inadequate immunization coverage (Roberts, 2018; Thompson and Kalkowska, 2019). Within days of vaccination, individuals shed pathogenic revertant viruses (Dunn et al., 1990) that can be transmitted to susceptible contacts. Furthermore, virulent cVDPVs circulate and persist for years in the environment and the community, often sub-clinically, providing a dangerous, "silent" reservoir of virus (Pallansch, 2018; Thompson and

CellPress

Kalkowska, 2019). The use of OPV can also result in vaccine-associated paralytic poliomyelitis (VAPP) in vaccine recipients and close contacts at an estimated rate of about 4.7 per million births (range: 2.4–9.7) globally (Platt et al., 2014).

Importantly, since elective Sabin2 vaccinations were stopped in 2016, most of the world has remained free of Sabin2 viruses. However, 12 distinct cVDPV2 outbreaks and over 50 VDPV2 events occurred after global cessation of routine use of Sabin2 from areas with persistently low immunization rates (Polio Global Eradication Initiative, 2020). Circulation of cVDPV2 constitutes a significant challenge to the eradication campaign and a major risk for global health, and has appropriately been designated as a Public Health Emergency of International Concern (Polio Global Eradication Initiative, 2020). On the other hand, IPV, an excellent tool to prevent paralytic poliomyelitis in vaccine recipients, is ineffective in preventing poliovirus transmission and combating epidemics in settings of poor hygiene and sanitation because it induces minimal intestinal mucosal immunity (Bandyopadhyay et al., 2015). The current response strategy to cVDPV2 outbreaks is to vaccinate with monovalent OPV type 2 vaccine (mOPV2), stockpiled for this purpose. This approach, however, can itself generate new cVDPV2 strains, as recently observed in parts of Africa (Blake et al., 2018; Jorba et al., 2018). The current situation highlights the need to develop and deploy live-attenuated vaccines as a tool to combat epidemics.

To confront the challenge of the reversion of OPV to cVDPVs, we sought to develop a rational approach to design a safer vaccine. We drew on ideas and knowledge from molecular and evolutionary studies of viral virulence determinants and mechanisms (Minor, 2012), as well our previous work, to understand to the molecular basis of attenuation and genetic instability of polio vaccines (Macadam et al., 2006). We also determined how mutations that accumulate during type 2 cVDPV epidemics enhance the replication fitness of poliovirus and increase virulence (Stern et al., 2017), thereby uncovering evolutionary pathways by which the Sabin vaccine strains become pathogenic. Additionally, we showed that adaptation and virulence are driven by the high error rate and recombination capacity of the viral polymerase (Vignuzzi et al., 2006; Xiao et al., 2017a, 2017b). We harnessed all of these insights to engineer a vaccine strain that preserves the antigenic and immunogenic characteristics of Sabin2 while increasing its safety by stabilizing determinants of attenuation. These features should lead to a safer and effective vaccine strain.

RESULTS

Engineering the Sabin Genome to Prevent Reversion to Neurovirulence

Given the past success of the Sabin2 vaccine, our objective was to preserve its biological properties and to prevent its reversion to neurovirulence by restricting its evolutionary capacity. Accordingly, in designing a live-attenuated vaccine, we preserved its overall replication strength and thermo-sensitivity characteristics, and thus its fitness in vaccinees and immunogenicity. The genome of this polio vaccine candidate (herein nOPV2) carries five modifications of the Sabin2 genome, including two modifications within the 5′-untranslated region (UTR) (relocated *cre* and S15domV), synonymous mutations at eight nucleotide positions in the 2C coding region to inactivate the internal *cre* (Figure 1A) and two mutations in the 3D polymerase (D53N and K38R) to limit viral adaptability (Figure 3A). Each of these modifications, described below in detail, contributes to genetic stability and attenuation. Importantly, their combination prevents detectable reversion to neurovirulence by reducing the capacity of the virus to acquire mutations that increase replication fitness in neuronal tissues.

Genetic Stabilization of the "Gate-Keeper" Attenuation Determinant, *domV*

The current type 2 vaccine is highly attenuated in both monkeys and poliovirus-susceptible mouse models (TgPVR mice). The major determinant of attenuation resides in the 5′ noncoding region (Macadam et al., 1992). This mutation (nucleotide 481A) weakens the thermostability of an RNA stem-loop structure, known as domain V (Sabin2 *domV*; Figure S1A). A single A-to-G mutation at this position results in increased tolerance to temperature and makes the virus more neurovirulent (Macadam et al., 1992, 1994; Stern et al., 2017). All three Sabin strains carry critical determinants of attenuation within *domV*. *DomV* forms part of the internal ribosome entry site (IRES), and it is thought that destabilizing this structure reduces the efficiency of translation initiation (Kauder and Racaniello, 2004; Macadam et al., 1994; Svitkin et al., 1990). OPV strains can revert to a form that causes paralysis in humans and can, under certain conditions, sustain circulation in the population. These viruses with regained virulence incorporate a number of mutations that increase the structural stability of *domV* (Contreras et al., 1992; Davis et al., 1977; Dunn et al., 1990).

Soon after vaccination, the majority of vaccinees shed polioviruses with a high frequency of such mutations (Evans et al., 1985; Stern et al., 2017), indicating that the structural stability of *domV* is under strong positive selection in the human gut. In all type 2 lineages in current outbreaks, the 481 A-to-G mutation preceded all other events leading to cVDPVs virulence (Stern et al., 2017), suggesting its role as a "gate-keeper" in the reversion process, similar to driver mutations in cancer evolution (Curigliano, 2018; Reddy et al., 2017; Temko et al., 2017). Accordingly, our approach was to maintain the sub-optimal thermodynamic stability of RNA *domV*, essential for the attenuation phenotype of Sabin2, but to manipulate the primary sequence in a way that prevents reversion by single-point mutations. Indeed, *domV* can be modified to alter viral virulence predictably and, at the same time, increase genetic stability to the extent that reversion is at undetectable levels (Macadam et al., 2006). The possibility that a single-point mutation can increase the overall thermostability of the structure is prevented by eliminating G-U base pairs, a strategy that ensures any single mutation must decrease stability, and hence fitness will be subject to negative selection. Other CG and/or UA base-pair exchanges can be used to maintain the reduced thermodynamic stability of the attenuating *domV* RNA structure in vaccine stains. The mechanism of attenuation in engineered strains is, thus, the same as that in Sabin strains, so the effects on "take" and growth in the human gut are expected to be minimal.

A genetically stabilized *domV*, S15, was originally constructed based on the idea described above on an OPV3 background. While this RNA domain is highly conserved at the structural level across poliovirus serotypes, there are 18 nucleotide differences between the S15 and Sabin2 *domV* sequences (Figure S1A, *i* and

A

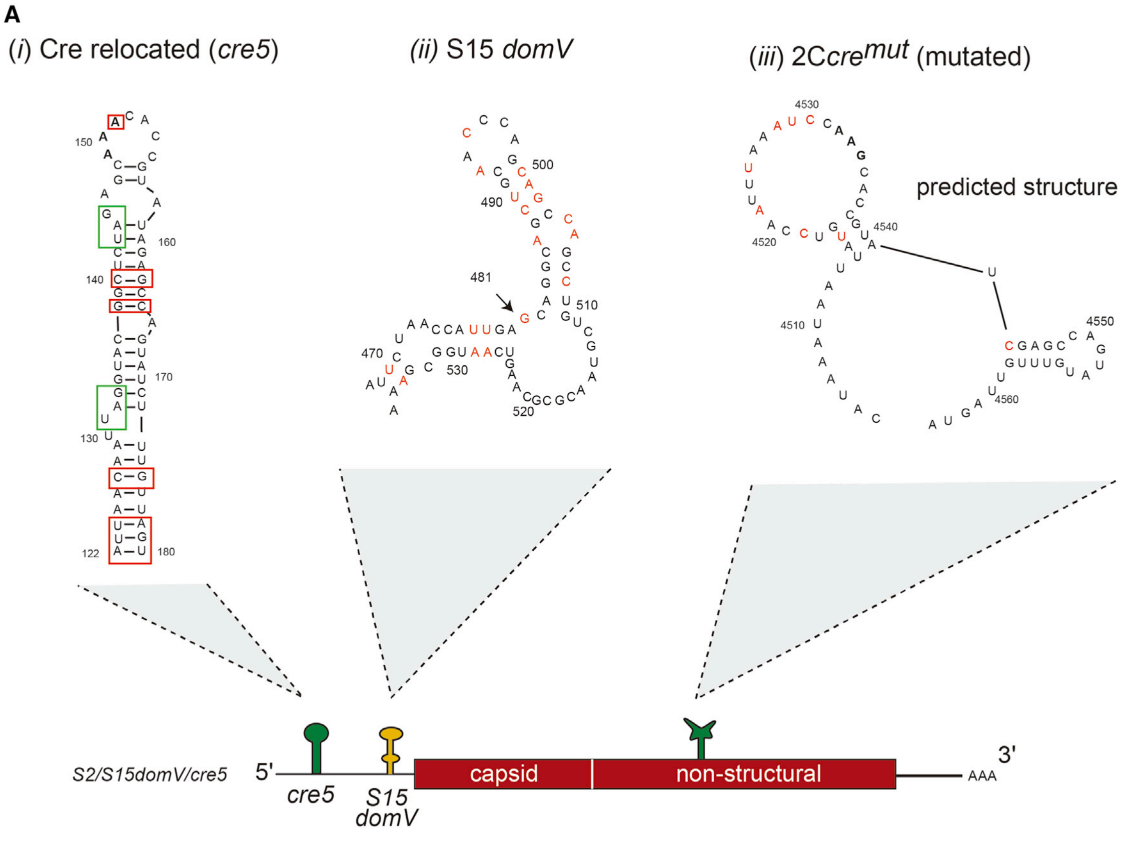

B Temperature sensitivity

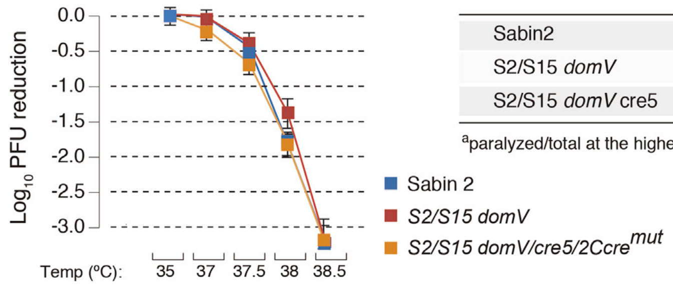

C Neurovirulence

	Tg66-CBA, i.s.
	PD$_{50}$ (log$_{10}$CCID$_{50}$)
Sabin2	5.9
S2/S15 *domV*	6.0
S2/S15 *domV* cre5	> 6.5 (0/8[a])

[a]paralyzed/total at the highest dose

Figure 1. Predicted RNA Secondary Structures of 5′-UTR *domV,* Relocated *cre* and Mutated Internal *cre*
(A) Schematic representation of the predicted RNA secondary structures of the following: (*i*) relocated *cre*, optimized base-pairs for temperature sensitivity and attenuation phenotypes are shown in red rectangles and "in-frame" STOP codons are shown in green rectangles; (*ii*) S15 *domV*, mutated bases to stabilize the RNA structure are shown in red; and (*iii*) mutated 2C-*cre* (2C*cre*^{mut}), shown as a predicted disrupted RNA secondary structure with nucleotide changes for 2C-*cre* function disruption are shown in red. Sabin2 carrying this structure was nonviable (data not shown).
(B and C) Temperature sensitivity (B) shown as mean with SD and neurovirulence (C) of these mutant viruses were evaluated in Vero cells and by intraspinal (i.s.) inoculation of poliovirus into susceptible Tg66 mice, respectively.

iii). To identify Sabin2 variants with growth and attenuation properties equivalent to those of Sabin2 but with greater genetic stability, Sabin2 was engineered with three versions of *domV* that differed in the proportion of the Sabin2 sequence substituted with that of S15 (Figure S1A). To examine the effects of these substitutions, we used a cell-culture assay that estimated temperature sensitivity, which is a characteristic correlating with attenuation in Vero cells (Macadam et al., 2006; Stern et al., 2017), and a neurovirulence test in susceptible transgenic mice (Stern et al., 2017). The temperature-sensitivity assay indicated that replacing the entire *domV* of Sabin2 with S15 had little or no effect on sensitivity at the tested temperatures (Sabin2 and

S2/S15 *domV* in Figures 1B and S1B, respectively), whereas replacement of just stems a and b (S2/S15 a, b *domV* in Figure S1B) or a, b, c, and d including the joining loops (S2/S15 *domV*, S2 loop in Figure S1B) produced viruses that were more temperature sensitive (*ts*) than Sabin2 (Figures 1B and S1B). The paralysis dose, 50% (PD_{50}) for each of these mutant viruses was also determined to better understand their effects on neurovirulence. Similar to the *ts* phenotype, replacement of the entire *domV* (Figure S1A, *iii*) had little effect on neurovirulence, whereas replacing S15 subdomains (Figure S1, *ii* and *iv*) further attenuated Sabin2 neurovirulence phenotype (Figures 1C and S1C). As we aimed to maintain the suboptimal thermostability of *domV* without further compromise virus fitness, we selected S15 *domV* (Figure 1A, *ii*).

Relocating 2C-*cre* Structure to Prevent nOPV2 *domV* Replacement via Recombination

Epidemiological studies on cVDPV outbreaks identified recombination as an important molecular mechanism that leads to increased viral fitness and virulence. Recombination of an OPV strain with co-circulating enterovirus C species allowed the Sabin2 *domV* to be replaced by that of a WT co-circulating enterovirus to provide a thermostable structure, thereby restoring viral fitness (Burns et al., 2013; Stern et al., 2017). If this were to occur with nOPV2, it would render our genetic modification of *domV* ineffective. We thus introduced an additional design element to prevent such a potential *domV* reversion through recombination. Briefly, we disrupted the secondary structure of the essential *cis*-acting replication element (*cre*) located within the 2C gene (Goodfellow et al., 2000; McKnight and Lemon, 1996) without changing the coding sequence (Figure 1, *iii*) and introduced a new *cre* within the 5′-UTR (untranslated region) upstream of *domV* (Figure 1, *i*, and 3A). The insertion of a new functional *cre* into the 5′-UTR between nucleotides 120 and 121 at the 5′-UTR yielded a poorly replicating, *ts* variant (*cre1*, Figures S2A, S2B, and S3A). We thus modified 5′ inserted *cre* by introducing mutations that increased thermostability of the structure at its new location. These modifications increased thermostability by converting U-G pairs into C-G pairs and by increasing the number of base-pairs at the bottom of the structure (Figure S2A, red boxes) and were able to rescue the poor replication ability of *cre1* (Figure S3A). Besides mutations increasing thermostability, two stop codons (Figure S2A, green boxes) were engineered into the new inserted *cre* to prevent generation of viable viruses via the relocated structure recombining back into its original 2C position. Accordingly, several *cre* candidate structures (Figure S2A, *Cre1a-5*) were constructed and examined with respect to their effect on replication and thermosensitivity. Engineered *5′cre* candidates tested in the presence of the S15 modification showed different levels of temperature sensitivity and reduced replication ability as compared with Sabin2 and S2/S15 *domV* (Figure S2B). Notably, relocation of *cre* further attenuated their virulence in the mouse transgenic model (Figure S2C). Among the new *cre* candidates, *cre5* restored replication ability and temperature sensitivity to levels comparable with that of Sabin2 (Figures S2B and S2C). Based on these results, we selected *cre5,* which is the most thermostable structure and maybe, therefore, genetically the most stable (Figure S2A). By relocating *cre* to the 5′ end of the genome, replacement of *domV* by recombination with a co-circulating enterovirus would require two consecutive recombination events at either side of *domV*, because a single event would also remove the *cre5*, resulting in loss of viability. By relocating the new *cre5* structure in proximity to *domV*, the frequency of such a double recombination event should be further reduced (see below as well as Figures 5 and S6).

Reducing Adaptation Capacity by Modifying the Viral Polymerase $3D^{pol}$

The concerted action of a high mutation rate and recombination are key to virus intra-host adaptation, spread through the infected individual and ultimately virulence (Vignuzzi et al., 2006; Xiao et al., 2017a). To further increase the genetic stability of nOPV2, we introduced two substitutions in the viral RNA-dependent RNA polymerase ($3D^{pol}$), which limit the adaptive capacity of the virus by reducing mutation rate and recombination frequency (Xiao et al., 2017a, 2017b). We used genetic screens to identify mutations that increase replication fidelity or reduce RNA recombination rate (Vignuzzi et al., 2006; Xiao et al., 2017a). High-fidelity Sabin2 variants were isolated by selecting mutagen-resistant variants as described (Pfeiffer and Kirkegaard, 2003; Vignuzzi et al., 2006). Briefly, HeLa S3 cells were infected with Sabin2 in the presence of 100 μM of ribavirin for 10 h. Virus was collected and used to infect fresh cells. A total of eight passages in 100 μM of ribavirin were performed to generate virus populations (Figure 2A). Highly accurate next-generation sequencing (CirSeq) was performed to identify beneficial mutations selected under mutagenic pressure as described (Acevedo et al., 2014). Eleven mutations were identified as beneficial in response to mutagen (ribavirin) selection (Figure 2B) and were engineered into the Sabin2 infectious cDNA clone for further comparison. To determine whether any of these mutations increase resistance to the mutagen, HeLa S3 cells were infected with engineered Sabin2 variants in the presence of various concentrations of ribavirin, and virus titers were determined with a standard plaque-forming assay. The mutation D53N showed significant resistance to the mutagen, when compared with Sabin2 (Figures 2C and S4), and it was selected for additional studies.

To identify recombination rate determinants, Sabin2 was engineered to express enhanced green fluorescent protein (eGFP). This Sabin2-eGFP recombinant virus is genetically unstable, in that homologous recombination results in deletion of the entire eGFP coding region (Figure 2D). However, variants with a reduced rate of recombination stabilize the eGFP-expressing viruses. Thus, GFP-positive viruses were selected after five rounds of passage in HeLa S3 cells, and five mutations were identified as potential recombination defective determinants by Sanger sequencing (Figure 2E). To determine whether any of the identified mutations indeed reduced the recombination rate, we modified a published CRE-Rep assay in which a replication incompetent replicon was used (Lowry et al., 2014). Instead of replicon, we constructed a Sabin2 carrying three stop codons at amino acids 38–40 of $3D^{pol}$ (S2d3D). *In vitro* transcribed RNA from S2dCre, S2dCre-*Rec1* to S2dCre-*Rec5*, and S2d3D were co-transfected into L929 cells (Figure 2F). Titers of the recombinant virus were determined with a standard $TCID_{50}$ assay (median tissue culture infectious dose). Results of the RNA co-transfection assay showed that mutations K38R, P243S, and L445M

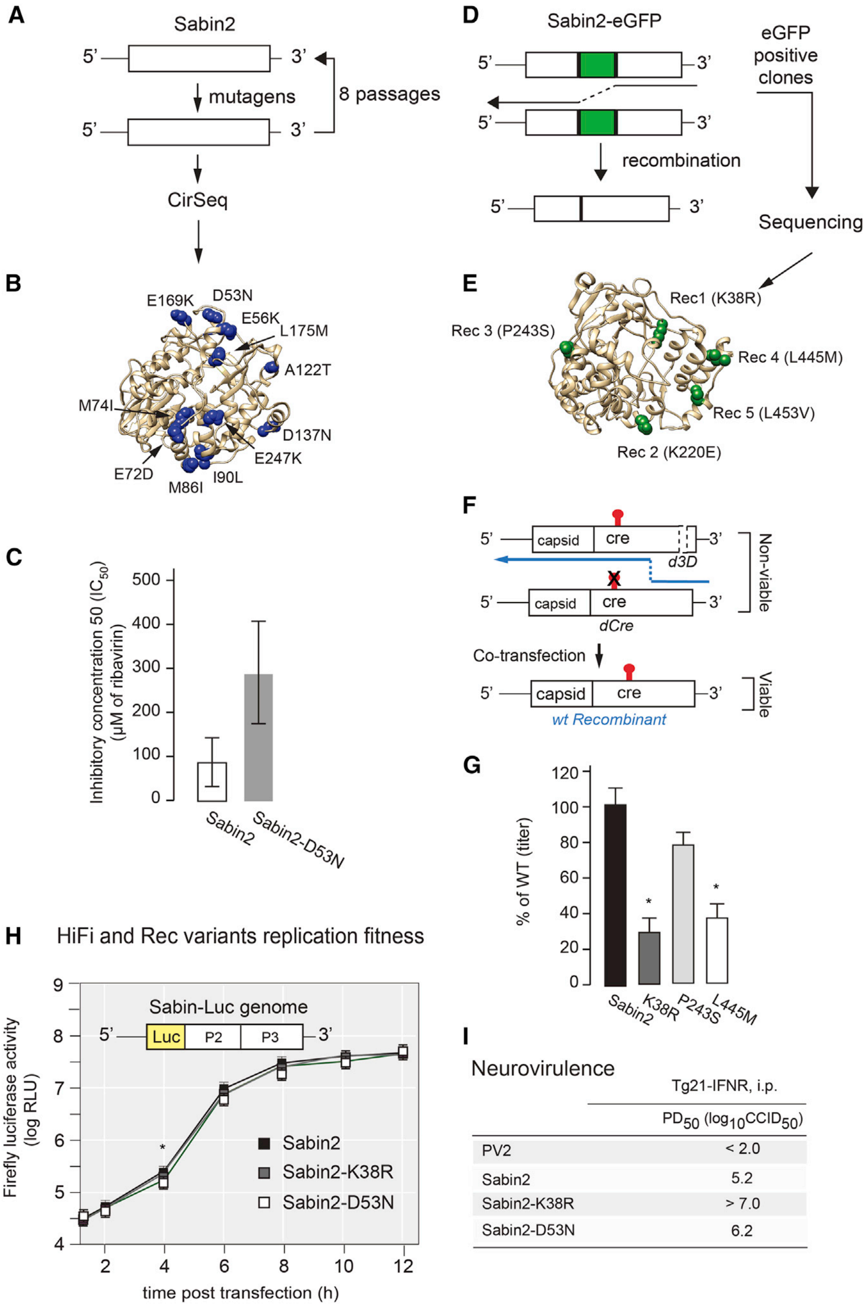

Figure 2. Modifications Were Introduced into $3D^{pol}$ to Increase Replication Fidelity and Reduce Recombination Rate

(A) To identify high-fidelity variants, Sabin2 virus was grown in HeLa S3 cells at m.o.i. of 0.1 with and without ribavirin under 33°C for 10 h. After eight passages, virus populations were analyzed by CirSeq (Acevedo et al., 2014) to identify fidelity determinants.

(B) Distribution of the 11 mutations revealed by CirSeq analysis as potential fidelity determinants on $3D^{pol}$ structure.

(C) To test for replication fidelity, we used a ribavirin-sensitivity test to determine the inhibitory concentration 50 (IC_{50}). Among the 11 fidelity candidates, *HiFi*3 (D53N) increased resistance the most to the mutagen ribavirin. The mean values calculated were significantly higher for Sabin-D53N than for Sabin2, with 95% confidence intervals that do not overlap.

(D) To identify recombination-rate determinants, the eGFP gene was inserted into the Sabin2 viral genome between P1 and P2 regions to generate Sabin2-eGFP virus. Serial passages of the Sabin2-eGFP virus often lead to eGFP loss because of homologous recombination, but variants with reduced recombination rates can be isolated by selecting eGFP-positive clones.

(E) After five passages in HeLa S3 cells, five potential recombination determinants were identified as shown on the $3D^{pol}$ structure.

(F) Experimental design of the "recombination validation assay." Viral RNAs with either mutated CRE or truncated 3D RNA polymerase do not produce viable viral progeny when transfected into L929 cells individually. Viable virus may be produced by recombination between the two truncated RNAs.

(G) Titers of viral progeny from the "recombination validation assay." Candidate mutations K38R, P243S, and L445M reduced recombination to 29.0%, 75.8%, and 38.7% to the parental strain Sabin2 (WT), respectively. Asterisk indicates significant difference as compared with WT; p values are 0.0322, 0.2277, and 0.0313 for K38R, P243S, and L445M, respectively. Data are shown as mean with SEM.

(H) Effect of *HiFi* (D53N) and *Rec1* (K38R) mutations on Sabin2 replication was initially determined using a luciferase replicon replication assay. Data are shown as mean with SEM. *p = 0.0187.

(I) Effect of *HiFi* (D53N) and *Rec1* (K38R) mutations on Sabin2 virulence was evaluated by PD_{50} of each mutant virus determined in PVRTg21 mice.

reduced recombination to 29%, 76%, and 37% of the parental strain. (Figure 2G). Mutation *Rec1* (K38R) was thus selected for additional tests.

We next characterized the effects of *HiFi* (D53N) and *Rec1* (K38R) on virus replication by introducing the two mutations into a Sabin2 replicon for replicon-luciferase assay and S2/S15domV/*cre5* for one-step virus growth analysis. These mutations have little effect on the kinetics of replicon replication (Figure 2H) and virus replication (Figure S5A). The PD_{50} for Sabin2 carrying *HiFi* (D53N) or *Rec1*(K38R) was determined in a mouse transgenic model, and both *HiFi* and *Rec1* further attenuated Sabin2 virulence (Figures 2I and S5C–S5F). We concluded that *HiFi* and *Rec1* attenuate Sabin2 virulence without impairing replication fitness (Figures 2H and S5A).

Replication Characteristics of nOPV2 Carrying Five Selected Modifications

A critical aspect of any live-attenuated vaccine is the ability to grow to high enough levels for production. Thus, we examined the fitness and replication properties of nOPV2 carrying all the

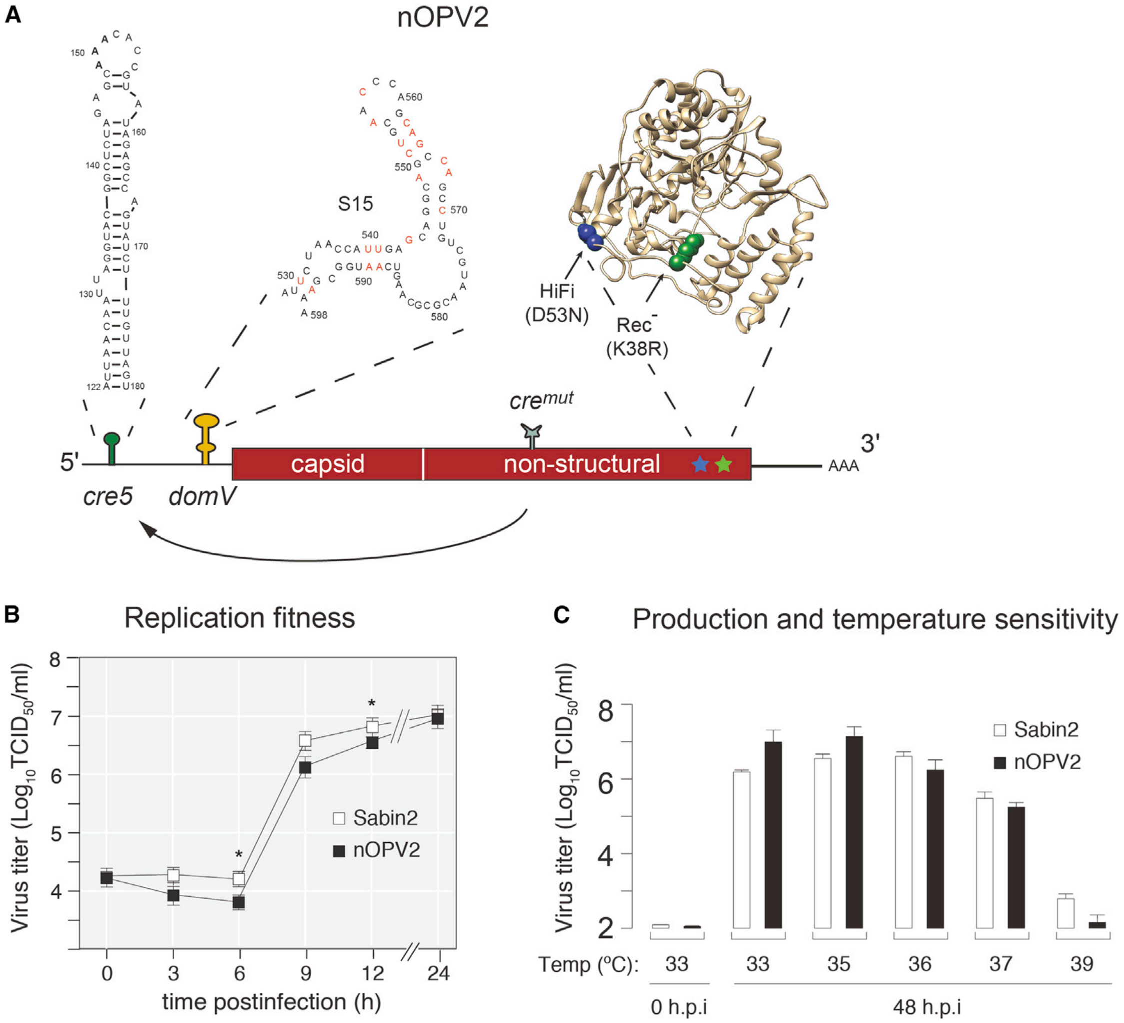

Figure 3. Sabin2 Vaccine Candidate (nOPV2) Design and Growth Phenotype in Cell Culture

(A) Schematic of the nOPV2 genome showing modifications and their locations. The sequence of 5′-UTR domain V (*S15 domV*) prevents an increase in *domV* thermostability by single-point mutations. To prevent replacement of *domV* attenuation elements by recombination, the *cre* element, essential for poliovirus replication, was relocated from its original position in the 2C coding region to the 5′-UTR (5′cre5). The original cre was inactivated by mutations (cre^{mut}); $3D^{pol}$ mutations *HiFi* (D53N) and *Rec1* (K38R) reduce overall virus adaptation capacity by reducing mutation and recombination rates, respectively.

(B) One-step growth analysis of nOPV2 and the current vaccine Sabin2 viruses in Vero cells (33°C) at m.o.i. of 10. Data are shown as mean with SEM. Asterisks represent statistically significant difference between Sabin 2 and nOPV2 production at 6 h ($p = 0.0080$) and 12 h ($p = 0.0197$) post-infection.

(C) Virus yield in Vero cells 48 h post-infection at different temperatures, m.o.i. = 0.01. Data are shown as mean with SD. Line and boxplots show mean with SD of triplicates.

modifications described above (Figure 3A) in Vero cells used for vaccine production (Neverov and Chumakov, 2010). Although lower virus titers were detected at 6 and 12 h post-infection from one-step growth analysis (Figure 3B), virus production and temperature sensitivity of nOPV2 under all tested temperatures were not weakened by the combined modifications, as compared with Sabin2 (Figure 3C). Importantly, because each modification in nOPV2 (*cre5*, S15 *domV*, *HiFi*-D53N, and *Rec1*-K38R) contributes independently to attenuation (Figures 1C, 2I, S2C, and S5C–S5F), their combination in one virus vaccine strain creates a multilayer safety-net that reduces the likelihood of nOPV2 to regain fitness and neurovirulence. Accordingly, reversion to a pathogenic virus from the vaccine will require the accumulation of multiple independent mutation events, including low probability AU-to-GC double mutations in the 5′ *Cre*, double recombination event in the 5′-UTR to replace S15 *domV* and remain viable, and reversion of two polymerase modifications that do not reduce replication fitness. A critical feature of this virus vaccine design is that nOPV2 maintains its replicative capacity in tissues without causing disease, which is essential to elicit a robust life-long immune response.

Genetic Stability of nOPV2

Next, we determined whether the re-designed nOPV2 is indeed more genetically stable than the current Sabin2 with a simple cell culture evolution paradigm (Figure 4A). The thermosensitive

A

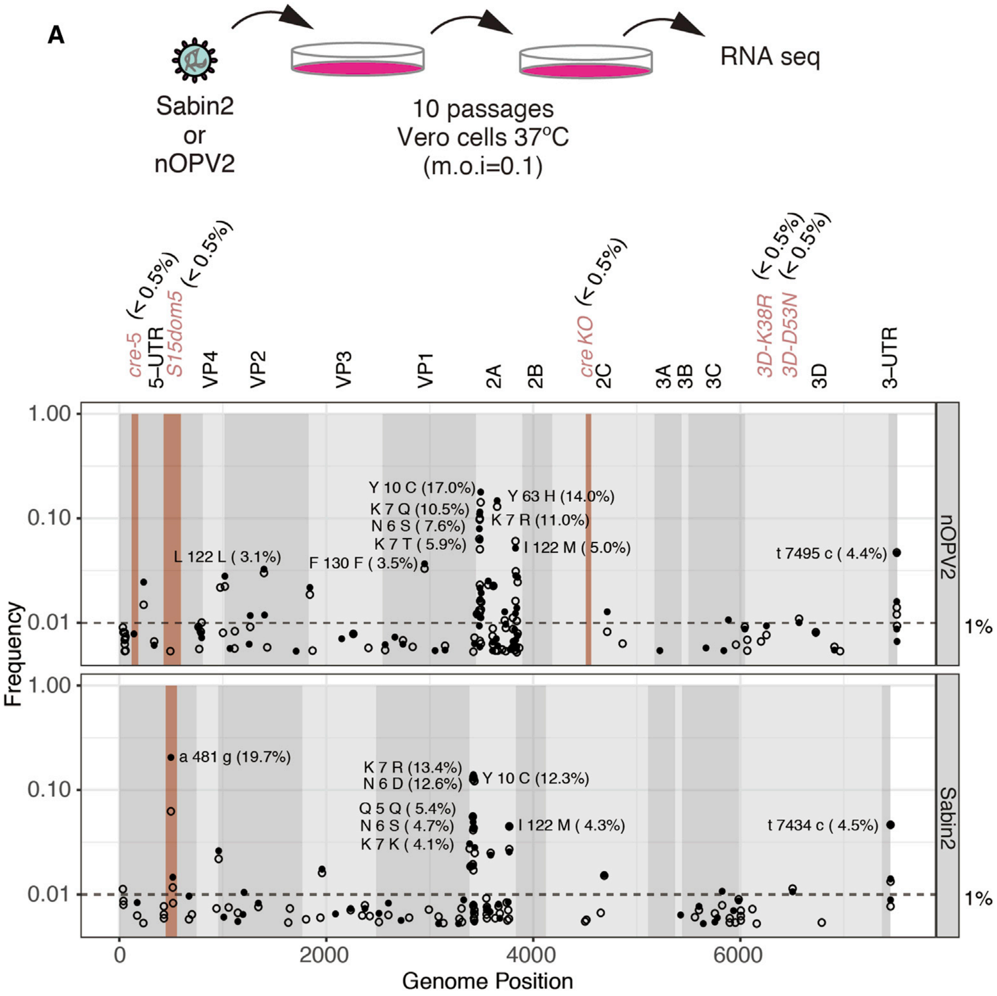

B

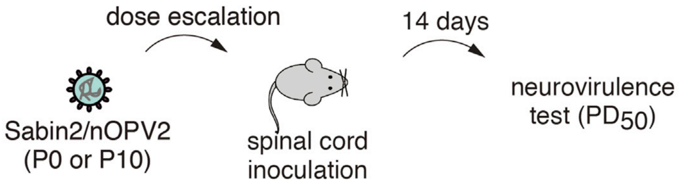

	PD50 (log CCID50)	
	P0	*P10*
Sabin2	5.9	3.9
nOPV2	> 8.35 (0/8[a])	> 8.7 (0/8[a])

[a]paralyzed/total at the highest dose

Figure 4. Genetic Stability of nOPV2 in Cell Culture and Animal Model of Infection

(A) Virus adaptation to high temperature in Vero cells. At the top, schematic of experimental design. Sabin2 and nOPV2 were grown in Vero cells at 37°C to accelerate virus evolution. After 10 passages, viral genomes of the 10 passage viruses (P10) were analyzed by RNA-seq. At the bottom, Manhattan plot showing

(legend continued on next page)

phenotype of Sabin2 is more pronounced in certain cell lines. For example, the serial passage of Sabin2 at 37°C in Vero cells, but not in Hep2C, promotes the rapid incorporation of mutations that increase both replicative fitness and thermotolerance (Manor et al., 1999). These thermoresistant variants also display a significant increase in virulence (Macadam et al., 2006; Stern et al., 2017). We thus carried out serial passage experiments using large viral population sizes and low multiplicity of infection (m.o.i.) (10^6 plaque forming units [PFUs] and m.o.i. = 0.1), that further ensures rapid evolution (Rouzine et al., 2001). Following 10 passages, we sequenced the virus population of both Sabin2 and the nOPV2 using next-generation sequencing to determine variant frequency. While the control Sabin2 vaccine strain acquired the neurovirulence determinant 481G within *domV* in approximately 20% of sequenced genomes, no nucleotide position with variant frequency of greater than 1% was identified within the regions of our five modifications (Figure 4A). In contrast, we observed accumulation of mutations, mostly in 2A region, at frequencies between 3%–17% in both Sabin2 and nOPV2 population (Figure 2A). These 2A mutations were reported to be associated with cell culture adaptation and have no effect on monkey neurovirulence (Rowe et al., 2000). Similar findings were obtained when the passages were carried out at a high m.o.i. (data not shown). Consistent with the increased genotypic stability of our engineered vaccine strain, the attenuated *ts* phenotype was retained by nOPV2 even after 10 passages at high temperature (data not shown).

We next examined virulence of nOPV2 with an intraspinal inoculation of transgenic mice expressing the human poliovirus receptor. This assay, used to test new lots of Sabin vaccines (https://www.who.int/biologicals/vaccines/), measures the PD_{50} (Figure 4B). The PD_{50} for Sabin2 in this model was determined as 5.9 log_{10} $CCID_{50}$ (50% cell-culture infective dose), whereas nOPV2 induced no paralysis in any mouse at the highest available dose up to 8.3 log_{10} $CCID_{50}$, indicating a significant decrease in neurovirulence as compared with Sabin2.

We also examined neurovirulence of populations of Sabin2 and nOPV2 subjected to high-temperature growth selection in Vero cells (Figure 4A). Consistent with the observation of accumulated 481G from next-generation sequencing, the high-temperature-adapted Sabin2 exhibited a PD_{50} of 3.9 log_{10} $CCID_{50}$, suggesting 100-fold greater neurovirulence than the original Sabin2. On the other hand, nOPV2 was attenuated with no measurable neurovirulence at the highest tested dose (Figure 4B). Thus, both deep sequencing and mouse neurovirulence testing confirmed that the designed vaccine candidate nOPV2 is more resistant to reversion to neurovirulence than Sabin2.

nOPV2 Does Not Exchange Its 5′-UTR by Recombination with Other Enteroviruses

Next, we tested the hypothesis that relocating the *cre* RNA stem-loop (Figure 5A) increases genetic stability by precluding replacement of the *domV* by recombination with a WT co-circulating enterovirus. HEp2c cells were co-infected under permissive conditions with Sabin2-derived variants and the closely related WT poliovirus type 3 (Leon strain, PV3), which is a potential donor of WT *domV*. To focus on the interplay of the redesigned *domV* and relocated *cre*, we compared Sabin2 control (Sabin2) and the Sabin2 variants with the *S15domV* only (*S2/S15domV*) or the combined *S15domV* and the relocated *cre* element in the 5′-UTR (*S2/S15domV/cre5*) (Figure 5A). PV3 virions were neutralized with serotype-specific polyclonal antibodies, and Sabin2-derived recombinants were selected by infection of L20B mouse cells at 37°C. The neutralizing antibodies prevented PV3 infection, and the L20B cells and high temperature favor the growth of viruses that had acquired a thermoresistant *domV* structure (Macadam et al., 2006; Stern et al., 2017). Co-infection in HEp2c cells was repeated up to five times to increase the probability of recombination between PV3 and Sabin2 variants. Potential recombinants of the three Sabin2 variants were examined after three rounds of selection in L20B cells at 37°C by RT-PCR (Figure S6B) or deep sequencing (Figure 5B). In agreement with previous reports (Burns et al., 2013; Stern et al., 2017), control virus Sabin2 readily acquired the 5′-UTR of PV3 containing a WT *domV* **(**Figures 5B and S6B**)**. The Sabin2 containing the *S15 domV*, but lacking the re-designed *cre5*, also acquired the 5′-UTR of PV3 containing a WT *domV* (Figures 5B and S6B). Strikingly, double modification in the *S2/S15domV/cre5* variant completely prevented recombination from acquiring the 5′-UTR of PV3, even after five rounds of co-infection and selection (Figures 5B and S6B).

These experiments also confirmed that the engineered S15*domV* had a lower probability of mutating to a thermostable structure. While we observed nucleotide 481 (A-to-G) revertant mutation in nearly 50% of the *domV* from Sabin2 control, neither S2/S15 nor S2/S15/cre5 variants acquired mutations within *domV* (not shown). Of note, our experimental design allowed the recovery of PV2/PV3 recombinants, as we detected recombination events for all type 2 variants downstream of nucleotide 5000 leading to acquisition of the non-structural coding region of the WT PV3 virus (Figure 5B). This event is often observed *in vivo* in vaccinees that receive trivalent OPV (tOPV) (Korotkova et al., 2017; Stern et al., 2017).

Deep sequencing of the selected recombinant viruses provided further details of their population's genetic structure. Approximately 40% of the Sabin2-derived viruses (Figure 5B, *i*) and approximately 70% of the *S2/S15*-derived viruses (Figure 5B, *ii*) inherited a WT *domV* from PV3, whereas the *S2/S15domV*/cre5-derived viruses all retained the S15*domV* (Figure 5B, *iii*); all reads mapped to type 2 in the capsid region (Figure 5B). Recombination events in Sabin2- and *S2/S15*-derived viruses were mapped at four sites, three between *domV* and the capsid proteins and one in VP4 that resulted in no coding changes. Single-nucleotide polymorphism analysis showed that 84% of the Sabin2-derived viruses that retained the Sabin2 5′-UTR had undergone A-to-G

frequency of mutations at several locations (*5′cre*, *domV, 2Ccre^{mut}, and HiFi/ Rec1*) in nOPV2, compared with those in Sabin2. In the *domV,* the frequency of A481G (19.7%) in Sabin2 is compared with any mutation that increases the thermostability of *domV* in nOPV2, which is a "gatekeeper" structure involved in regain of virulence. Open circles represent frequencies of mutations observed at passage 8 and solid circles at passage 10.

(B) Genetic stability was further validated by comparing PD_{50} of Sabin2 and nOPV2 before (P0) and after 10 passages (P10) of accelerated evolution at higher temperature as shown in (A). PD_{50}s of Sabin2 and nOPV2 (P0 and P10) were determined by intra-spinally inoculating Tg66 mice and calculated using the Spearman-Karber method.

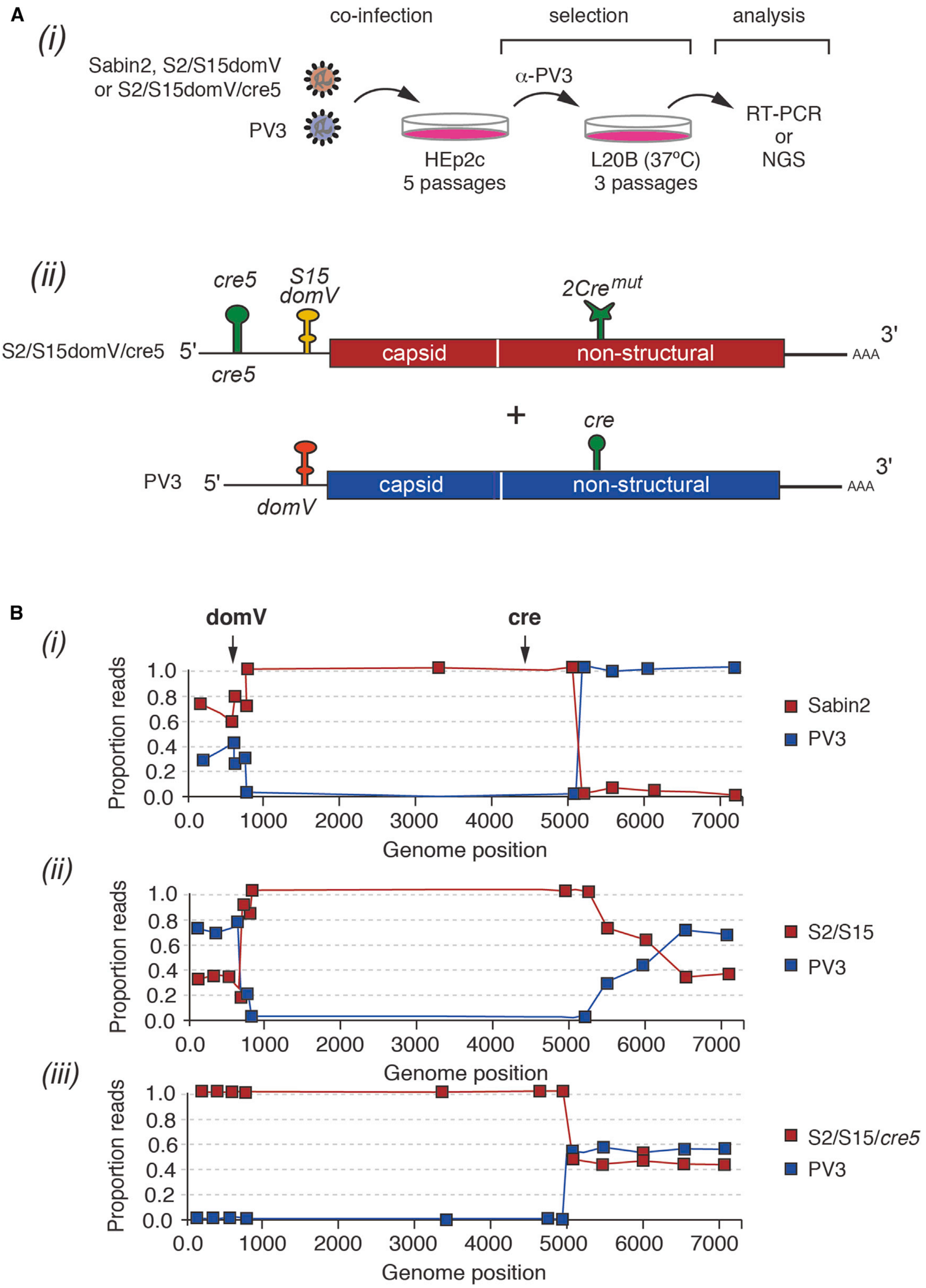

Figure 5. Preventing 5′-UTR Exchange by Recombination with Co-circulating Enteroviruses

(A) Schematic of recombination assay (*i*). Sabin2 or nOPV2 strains were used to coinfect HEp2C cells together with poliovirus type 3 (PV3). After co-infection, type 2 poliovirus recombinants were selected using antibodies directed against poliovirus type 3 and two rounds of replication in L20B at 37°C that selects for high fitness, temperature-resistance variants. Co-infection in HEp2c was repeated five times to increase the chance of recombination. Type 2 strains tested (*ii*) were as

(legend continued on next page)

mutation at nucleotide 481, the site of the major attenuating mutation in Sabin2. No mutations were observed in the *domV* of S2/S15domV-derived viruses that retained the S15*domV* or the S2/cre5/S15*domV*-derived viruses. Importantly, the attenuation phenotype determined as PD_{50} was preserved in S2/S15*domV*/cre5-derived viruses but lost in the Sabin2 and S2/S15 groups (Figure S7). As a result of the genetic changes in the 5′-UTRs of Sabin2 and S2/S15*domV* after co-culture with PV3 Leon, both populations reverted to a neurovirulent phenotype, as shown by their PD_{50} (1.8 log_{10} $CCID_{50}$, similar to S2/481G, Figure S7) representing a >4 log_{10} increase in virulence when inoculated intraspinally into TgPVR mice. The equivalent population derived from S2/S15*domV*/cre5 was as attenuated as Sabin2 (PD_{50} = 5.6 log_{10} versus 5.9 log_{10} for Sabin2; Figures 1 and S7). These results indicate that these modifications prevent reversion either by the acquisition of mutations or recombination, in support of our original hypotheses.

Antigenic and Immunogenic Characteristics of nOPV2

Because the nOPV2 strain is intended to elicit immune protection against virulent poliovirus strains, we analyzed the antigenic structure of the vaccine candidate in an ELISA assay similar to that used to measure the D-antigen content of poliovirus vaccines. A panel of monoclonal antibodies (mAbs), specific for native conformations of antigenic sites 1 (mAb 433), 2a (mAbs 1247), 2b (mAb 1037), and 3b (mAbs 1050), were used as primary antibodies (Minor et al., 1993). The WHO SO+2/2 (OPV2) strain was used as a reference in each experiment. Differences in antigenicity were not noted at any of the four sites tested (Figure 6A).

We then carried out an initial evaluation of nOPV2 immunogenicity. Susceptible transgenic mice were infected intraperitoneally with dilutions of Sabin2 or nOPV2, and serum samples were collected at 21 days post-inoculation and tested by neutralization (NT) assay to determine antibody titers. Inoculation with Sabin2 tended to induce higher geometric mean titers of neutralizing antibodies, particularly at low doses (Figure 6B). However, the difference between antibody titers induced by Sabin2 and nOPV2 at the tested doses and at the tested sample size did not reach statistical significance (Figure 6B, two-tailed Mann-Whitney U test). Importantly, Sabin2 and nOPV2 had similar seroconversion rates, 75%–100% of vaccinated mice generated neutralizing antibodies at the tested doses with a single immunization (Figure 6B, numbers on the top of the graphic). Seroconversion is a measure of how well a vaccine “takes” because it depends on virus replication as ultraviolet inactivated preparations are not immunogenic (Figure 6B, Sabin2 [UVI]). Hence the immunogenicity of nOPV2 was not significantly inferior to that of Sabin2. Additional experiments in humans will be required to further establish the immunogenic capacity of nOPV2.

Evaluating nOPV2 in Humans Reveals Its Potential as an Effective and Safer Vaccine

Based on these encouraging pre-clinical results, a phase I human clinical trial was previously performed to determine if nOPV2 is safe, immunogenic, and genetically stable as described in Van Damme et al. (2019). This phase I trial was conducted in 15 adult volunteers, previously immunized with inactivated poliovirus vaccine (IPV). Each volunteer received 10^6 $CCID_{50}$ of virus by oral administration (Figure 7A). Analysis of shed virus in stool samples showed effective intestinal mucosal replication of the vaccine virus in all vaccinees. Virus shedding in stool was observed in 14 collected samples at day 7 post-administration and in five of 11 collected samples on day 28. Importantly, vaccination with nOPV2 elicited a significant increase in neutralizing antibody titers against type 2 poliovirus in at least 83% of the subjects (Van Damme et al., 2019). An anamnestic antibody response was evident by an increase in the median neutralizing antibody titer from 56.9 (interquartile range [IQR] with 40.6–147.4 as Q1–Q3) before vaccination to 1,152.0 (IQR with 650.0–1448.0 as Q1–Q3) at 28 days post-vaccination (Van Damme et al., 2019). This represents a boost in neutralizing antibodies titers of more than eightfold (confidence interval, 2.0–20.1). Strikingly, in the mouse neurovirulence test, the redesigned nOPV2 recovered from vaccinees maintained its attenuation phenotype (Van Damme et al., 2019), in contrast to the current vaccine containing Sabin2 (Stern et al., 2017). While virus shed by Sabin mOPV2 vaccinees isolated 7 days after vaccination caused paralysis in 90% of the mice in a neurovirulence assay (Asturias et al., 2016), the virus shed by the nOPV2 vaccinees showed minimal paralysis rates (mean 1.6%, range 0%–10.0% for the 15 samples) at the same dose. This suggests that the modifications introduced into the nOPV2 enhance its genetic stability, which results in a significant reduction in reversion to neurovirulence (Van Damme et al., 2019).

Characterization of the Shed Viruses

To further examine the genetic characteristics of the vaccine strain, the viruses isolated from stool samples from the vaccinees were analyzed by deep sequencing to determine their evolutionary pathways in the human gut (Figure 7A). Multiple previous studies of Sabin2 vaccination reveal that within days post-vaccination, the virus accumulates mutations at position 481. Indeed, by 14 days post-vaccination, most Sabin2 vaccinees excrete virus mutants that lost the attenuation allele entirely in *domV* (Figures 7A and 7B) (Stern et al., 2017). In striking contrast, the individuals vaccinated with the nOPV2 excrete virus that has no mutations at any position in *domV* (Figures 7A and 7B). Several mutations were found in the nOPV2 virus shed by vaccinees. We used Shannon’s entropy calculation to determine what regions of the genome were altered during replication in vaccinees (Figure 7B). Substitutions at three positions were present at significant frequencies in viruses excreted by several subjects after about 2 weeks of replication: variation at VP1-143, the secondary attenuation site in Sabin2 (Macadam et al., 1994), and nucleotide 459 (U-C) within IRES domain IV (*domIV*) were comparable with that for Sabin2 in a previous study (Stern et al., 2017); mutations U123C or G179A at the base of the 5′ *cre* stem were also observed. Structural analysis suggests that

follows: Sabin2; S2/S15 *domV*, which does not revert by single point mutations, but potentially can acquire a more thermostable *domV* by recombination with PV3; and S2/S15 *domV*/*cre5*, in which 2C *cre* has been relocated into the 5′-UTR.
(B) After five rounds of co-infection and L20B selection, virus populations were sequenced, and the proportion of reads corresponding to PV3 or Sabin2 variants was calculated across the genome. Recombination between PV3 and (*i*) Sabin2, (*ii*) S2/*S15*, and (*iii*) S2/*S15*/*cre5*.

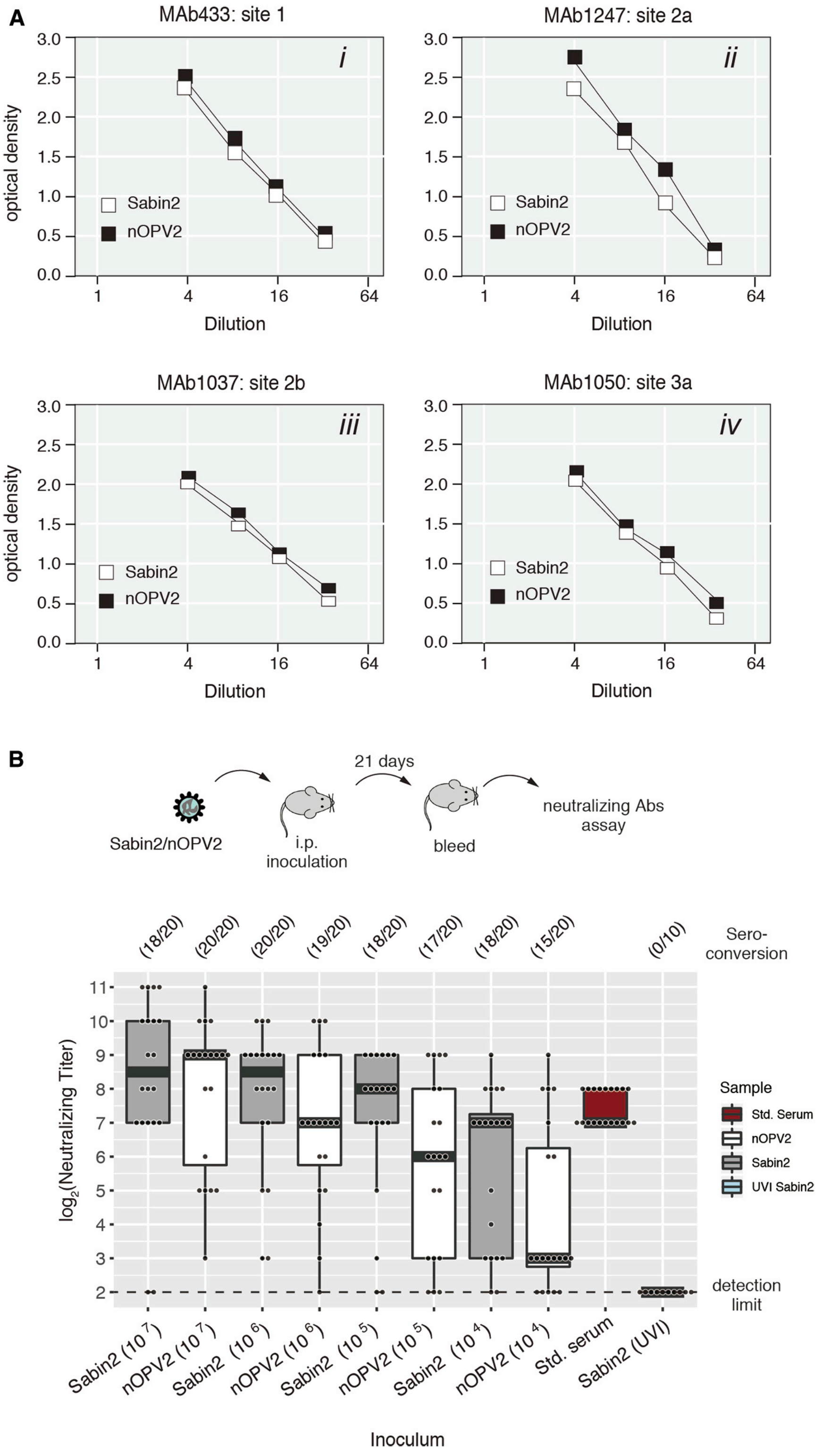

Figure 6. Antigenicity and Immunogenicity of nOPV2 in Mice

(A) Reactivities of nOPV2 with monoclonal antibodies against four antigenic sites on the poliovirus type 2 virion were compared to Sabin2 reactivity in ELISA assays.

(B) Interferon-receptor knockout, transgenic mice expressing the human poliovirus receptor (IFNAR−/− TgPVR21) were inoculated intraperitoneally with a range of doses (10^4–10^7 PFU) of Sabin2 and nOPV2. Mice injected with 10^7 pfu of ultraviolet light inactivated (UVI) Sabin2 were included as controls. Ten mice were used per condition for Sabin2 and nOPV2 and five mice for Sabin2 (UVI) in each experiment. Data shown were collected from two experiments. Titers of neutralizing antibody (NT) in sera at day 21 were determined by NT assay as described in STAR Methods. Box-and-whisker diagram represents the neutralizing antibody response for each condition. Bars in boxes represent median antibody titers. Whiskers represent the range of non-outliers observations (less than 2.5* IQR from the median). Overlapping dots represent neutralizing antibodies values obtained for each individual mouse. Statistical analysis (two-tailed Mann-Whitney U test) was performed to compare difference in titers of neutralizing antibody induced by Sabin2 and nOPV2: (p value: 0.53707, 0.1564, 0.0775, and 0.0731 for 10^7, 10^6, 10^5, and 10^4, respectively). Std is serum from a human subject vaccinated with OPV. At the top of the graph, seroconversion frequency (number of individuals that seroconverted over total).

U123C and G179A would strengthen the 5′cre by converting a U-G base pair at the base of the structure into a C-G or U-A base-pair (Figure S2). Mutation U459C (nucleotide 398 in Sabin2) was identified as a secondary adaptive mutation in cVDPV2 isolates and vaccinee isolates (Stern et al., 2017). We also observed additional mutations within the capsid proteins (Figure 7B, VP4-41 and VP1-33), but these mutations were not consistently present in all isolates, and we did not conduct further analyses on them. Based on the sequencing analysis, on average 7%–70% of shed virus isolated around 2 weeks post-vaccination had U123C/G179A, U459C, or VP1-I143T individual mutations. Thus, only a small proportion of shed viruses would carry all three of these mutations. Only after 8 weeks post-vaccination was an individual found to be shedding viruses that had fixed all three modifications (data not shown).

To determine if the mutations observed in the virus shed from vaccinees could increase their fitness and virulence, we engineered these mutations into the infectious cDNA clones corresponding to these viruses. We introduced into nOPV2 genome substitutions U123C (5′ *cre)*, U459C (*domIV*), and A2969G (VP1-I143V) into nOPV2 genome to generate an nOPV2 "triple mutant" (nOPV2) (Figure 7C). We also cloned A481G (*domV*) into the Sabin2

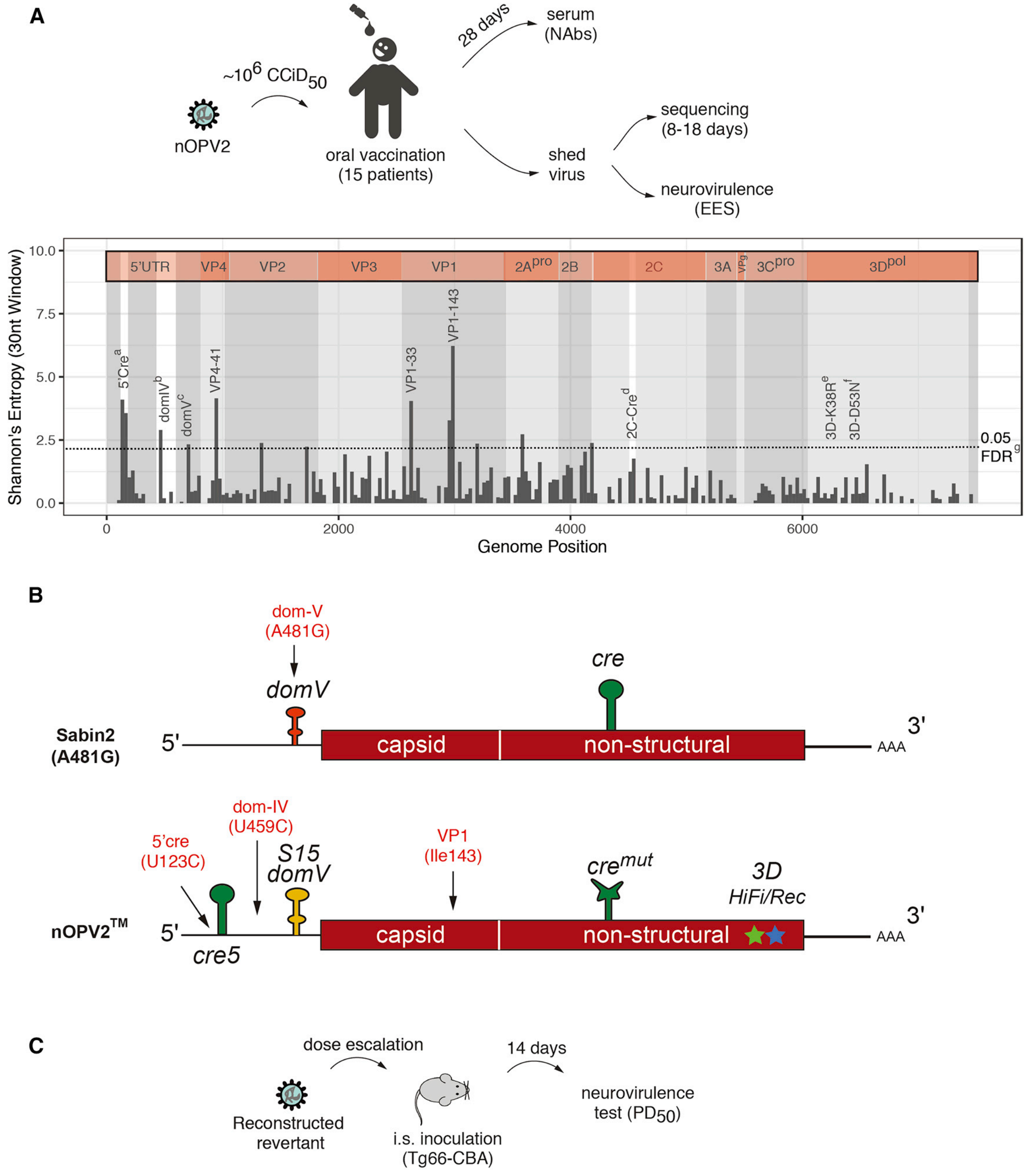

	PD_{50} (log $CCID_{50}$)
Sabin2	5.9
Sabin2 (A481G)	1.9
nOPV2	> 8.35 (0/8[a])
nOPV2™ (U123C, U459C and I143V)	4.3

[a]paralyzed/total at the highest dose

Figure 7. Genetic Stability of nOPV2 in Human Volunteers

(A) Top panel: schematic of vaccine administration and analysis in humans. Fifteen volunteers were inoculated with nOPV2 by oral route with 10^6 cell-culture infectious doses 50% ($CCID_{50}$). Viruses were isolated from stool samples at 8–18 days post-vaccination. Mean and range of variant frequencies observed in samples from days 8 and 18 for nOPV2 in this trial are compared with data obtained previously for Sabin2 at 14 days post-vaccination with trivalent OPV (tOPV in

(legend continued on next page)

(Sabin2/A481G). The reconstructed nOPV2 (U123C, U459C, and VP1-I143V) virus is the "worst-case" scenario in which all the mutations that were selected in shed viruses after many weeks of replication had accumulated to 100% in a single genome, imparting the maximum increase of fitness (Stern et al., 2017). Sabin/A481G represents the *de facto* case when Sabin2 is employed (i.e., a revertant virus excreted by most vaccinees within days of vaccination). We then evaluated neurovirulence of the reconstructed viruses in transgenic mice after intra-spinal inoculation. Introduction of A481G alone into the Sabin2 genome produced a neurovirulent virus with a PD_{50} of 1.9 (log $CCDI_{50}$), and the mutant nOPV2 had a PD_{50} of 4.3 (Figure 7C). Thus, even in the unlikely event that nOPV2 accumulated all three changes in one genome, the strain would be at least 250 times more attenuated than the single-mutation Sabin2 revertant (Sabin2/481G), which is produced almost universally days after vaccination.

DISCUSSION

Here we describe the development of an attenuated poliovirus vaccine candidate, nOPV2, and show it possesses the fitness and immunogenicity of Sabin2, but, importantly, is genetically more stable than the current Sabin2 strain. Our approach was to leverage decades of basic research on the biology of picornaviruses into a rational design of a live-attenuated vaccine with improved safety and stability. Five modifications were introduced in the Sabin2 viral genome, based on our understanding of viral biology and pathogenesis. Our results from cell culture (Figures 1B, 2H, 3B, 3C, 4A, 5B, and S5A), small animal models (Figures 1C, 2I, 4B, S1C,S2C,S5C–S5F, and S7), and an initial clinical trial (Figure 7) all demonstrate that the concerted action of these modifications produces a vaccine candidate strain that, while replicating at similar levels as Sabin2, is less likely to evolve into a neurovirulent cVDPV or cause vaccine-associated paralytic poliomyelitis. As oral live attenuated poliovirus vaccines are required to interrupt person-to-person transmission during outbreaks of the kind the world is currently witnessing (Bandyopadhyay et al., 2015; Manor et al., 1999), this strain may facilitate the control of poliomyelitis outbreaks around the world.

Our strategy introduced modifications in the 5′-UTR that prevent the reversion of a major "gatekeeper" attenuation determinant within *domV*. In Sabin2 a transition mutation therein, A481G, is rapidly fixed upon replication in vaccinees (Neverov and Chumakov, 2010). Mutations at other positions in domain V are also strongly selected in Sabin1 and Sabin3 vaccines, and this is driven by increases in the RNA secondary structural stability of this domain (Macadam et al., 1992). Temperature sensitivity and attenuation phenotypes of mutants with defined changes in domain V correlated well with predicted secondary-structure stabilities. Reversal of base-pair orientation had little effect, whereas stem disruption increased temperature sensitivity and revertants displayed mutations on either side of the stem. These observations suggested the strategy used in this study to stabilize attenuation, namely an engineered *domV* with an overall structural stability (ΔG) similar to that of attenuated Sabin strains, but using base-pairs that cannot increase stability by a single-point mutation (Macadam et al., 2006). We observed no mutations accumulating in *domV* in cell-culture selection experiments (Figure 4A) or after replication in humans (Figure 7A), indicating that the modifications introduced in *domV* in nOPV2 prevent the rapid evolution of this element.

We also relocated *cre* from its normal 2C location to the 5′-UTR to prevent the exchange of the entire nOPV2 *domV* with that from a co-circulating enterovirus C, such as PV3. After five rounds of co-infection in Hep2C cells, followed by extreme selection conditions (replication at 37°C in L20B cells), nOPV2 did not replace *domV* by recombination. The relocated *cre5* is close to *domV*, which is expected to reduce the recombination rate. Also, the probability of recombining out the modified 5′-UTR is even lower, given that the substitution of *domV* requires two simultaneous recombination events (Figures 5 and S6).

Two other modifications were introduced into viral RNA polymerase of nOPV2. The rapid adaptation of RNA viruses, which is linked to virulence (Vignuzzi et al., 2006; Xiao et al., 2017a), is enabled by virus population diversity, which increases the probability of accumulating beneficial and adaptation mutations (Domingo and Holland, 1994; Domingo et al., 2006; Lauring and Andino, 2010). Such genetic diversity, driven by the error-prone virus polymerase, provides an opportunity for individual beneficial alleles to be selected in a given host to circumvent environmental challenges, such as antiviral immune responses. However, diversity comes at a cost, because most mutations have deleterious effects. Thus, selection of beneficial mutations requires breaking the linkage between detrimental and beneficial mutations (Chao, 1990; Duarte et al., 1992) and avoiding competition between beneficial mutations ("clonal interference" effect) (Fisher, 1930; Lenski et al., 1998; Roze and Barton, 2006; Worobey and Holmes, 1999). Recombination tends to oppose these negative effects by bringing beneficial mutations into the same genome and purging deleterious mutations (Worobey and Holmes, 1999). This would allow putative beneficial alleles to be selected in a given host environment. Indeed, viral populations with reduced adaptation capacity yield viruses that are tissue restricted, and thus attenuated (Xiao et al., 2017a; 2016). One of our mutations enhanced the accuracy of the viral polymerase by increasing the fidelity of replication (*HiFi*,

polio vaccine naive human subjects; Stern et al., 2017). Bottom panel: to highlight regions of diversity across individuals, we used full-genome sequences obtained from all EES samples to calculate the sum of Shannon's entropy across vaccines along the genome in 30-nucleotide windows. 5′Cre[a] (U123C or G179, mean: 0.68; range: 0.19–1.00), *domIV*[b] (nt459, mean: 0.07; range: 0.00–0.40), *domV*[c] (any mutation UA to CG that increases *domV* structural stability, mean: <0.005, the limit of detection); 3D[e & f] (any mutation in $3D^{pol}$ at positions HiFi K38R or Rec D53N, mean: <0.005). [g]95% extreme value test from 10,000 randomly sampled 30mers. We also observed significant increase in Shannon's entropy in capsid proteins VP4 (position 41) and VP1 (positions 33 and 143).

(B) Positions for the most frequent mutations identified from shed viruses: *domV* A481G in Sabin2 (Evans et al., 1985); U123C (5′ *cre*), U459C (*domIV*), and A2969G (VP1-I143V) in nOPV2.

(C) Schematic representation for virulence test of reconstructed viruses (top panel). Mutations accumulating at high frequencies in virus isolated from vaccinees (B and Stern et al., 2017) were engineered into infectious molecular clones of Sabin2 or nOPV2 to generate Sabin2 (A481G) and nOPV2 (U123C, U459C, and I143V), respectively. Virulence (PD_{50}) of reconstructed viruses was determined with the Tg66-CBA mouse model after intraspinal inoculation (bottom panel).

D53N), and the second reduced the rate of recombination (Rec1, K38R). Of note, either mutation individually or both in combination supported efficient virus replication in cell culture (Figures 2H and S5A). However, each further attenuated virulence of Sabin2 in transgenic mouse model (Figures 2I and S5B–S5F). These observations support previous experiments indicating that the diverse genetic composition of an RNA virus population is a virulence determinant that allows the virus to navigate dynamic environments.

The nOPV2 strain developed here appears to be more genetically stable than Sabin2. However, additional studies must examine the potential for the reversion of nOPV2. Longer timescales and a large population size will be needed to evaluate the long-term safety of the vaccine strain further. It will be very important to determine the phenotypic and genotypic characteristics of nOPV2-derived viruses isolated from individuals infected with the vaccine strain indirectly by contact with vaccinees.

Candidate vaccines that incorporate design elements coming from the molecular and epidemiological understanding of virus biology, such as the nOPV2 developed here and other alternative vaccine candidate designs developed in the context of our Bill and Melinda Gate Foundation Consortium (Konopka-Anstadt et al., 2020), should be a game-changer in the efforts to contribute to the complete and permanent global eradication of poliovirus type 2. Application of similar knowledge-based strategies to vaccine design should also enable us to rationally design live-attenuated vaccines for other polio-serotypes as well as related emerging pathogenic enteroviruses, including enterovirus D68 (EV-D68) and enterovirus A71 (EV-A71).

STAR★METHODS

Detailed methods are provided in the online version of this paper and include the following:

- KEY RESOURCES TABLE
- RESOURCE AVAILABILITY
  - Lead Contact
  - Materials Availability
  - Data and Code Availability
- EXPERIMENTAL MODEL AND SUBJECT DETAILS
  - Cells and Viruses
  - Mouse Model
  - Human Subjects
- METHOD DETAILS
  - Relocation and Optimization *of cre*
  - Selection of *HiFi* Mutations
  - Ribavirin Resistance Assay
  - Construction of Sabin2-eGFP Virus Infectious Clone
  - Selection of Recombination Deficient Virus
  - Generation of Sabin2-*HiFi* and Sabin2-*Rec1* Viruses
  - Construction of Sabin2 Replicon and 3D/CRE Truncation
  - Recombination Validation Assay
  - Replicon Replication Assay
  - Construction of nOPV Variants
  - nOPV2(S2/cre5/S15domV/Rec1/HiFi3)
  - Virus Recovery
  - Virus Growth Analysis
  - Virus Passages under Accelerated Evolution
  - Next-Generation Sequencing
  - Neurovirulence Test in Tg66 Mice (TgmNVT)
  - Recombination Assay
  - Immunogenicity Assay
  - Antigenicity Assay
- QUANTIFICATION AND STATISTICAL ANALYSES

SUPPLEMENTAL INFORMATION

Supplemental Information can be found online at https://doi.org/10.1016/j.chom.2020.04.003.

ACKNOWLEDGMENTS

This work was carried out as part of the nOPV2 Consortium, funded by the BMGF, which also included Cara Burns, Olen Kew, Konstantin Chumakov, and Jennifer Anstadt. We thank them, current, and former advisors (Ellie Ehrenfeld, Peter Wright, John Modlin, Jacqueline Fournier, and Martin Eisenhauer) for highly productive discussions throughout this collaboration. We further thank Judith Frydman, Orly Laufman, and Michel Tassetto for helpful discussions and comments on the manuscript, and Sarah Knowlson and the NGS and BSD teams at NIBSC for technical assistance. This work was supported in part by NIH (R01 AI36178, AI40085, P01 AI091575), the Department of Health Policy Research Programme (NIBSC Regulatory Science Research Unit, 044/0069), and the Bill and Melinda Gates Foundation.

AUTHOR CONTRIBUTIONS

M.T.Y., A.M., and R.A. designed the project. A.M. and R.A. contributed to conceptualization; M.T.Y., E.B., and M.S. contributed to investigation; R.W., J.K., P.V.D., I.D.C., A.J.W., A.S.B., and H.R. were involved in setting up and conducting the phase 1 clinical trial, and the analyses of the clinical data; M.T.Y., P.T.D., A.M., and R.A. contributed to the analyses, interpretation, and writing of the results.

DECLARATION OF INTERESTS

The authors declare no competing interests.

Received: June 2, 2019
Revised: December 3, 2019
Accepted: April 2, 2020
Published: April 23, 2020

REFERENCES

Acevedo, A., Brodsky, L., and Andino, R. (2014). Mutational and fitness landscapes of an RNA virus revealed through population sequencing. Nature *505*, 686–690.

Asturias, E.J., Bandyopadhyay, A.S., Self, S., Rivera, L., Sáez-Llorens, X., Lopez, E., Melgar, M., Gaensbauer, J.T., Weldon, W.C., Oberste, M.S., et al.; Latin American IPV001BMG Study Group (2016). Humoral and intestinal immunity induced by new schedules of bivalent oral poliovirus vaccine and one or two doses of inactivated poliovirus vaccine in Latin American infants: an open-label randomised controlled trial. Lancet *388*, 158–169.

Bandyopadhyay, A.S., Garon, J., Seib, K., and Orenstein, W.A. (2015). Polio vaccination: past, present and future. Future Microbiol. *10*, 791–808.

Blake, I.M., Pons-Salort, M., Molodecky, N.A., Diop, O.M., Chenoweth, P., Bandyopadhyay, A.S., Zaffran, M., Sutter, R.W., and Grassly, N.C. (2018). Type 2 Poliovirus Detection after Global Withdrawal of Trivalent Oral Vaccine. N. Engl. J. Med. *379*, 834–845.

Burns, C.C., Shaw, J., Jorba, J., Bukbuk, D., Adu, F., Gumede, N., Pate, M.A., Abanida, E.A., Gasasira, A., Iber, J., et al. (2013). Multiple independent

emergences of type 2 vaccine-derived polioviruses during a large outbreak in northern Nigeria. J. Virol. *87*, 4907–4922.

Burns, C.C., Diop, O.M., Sutter, R.W., and Kew, O.M. (2014). Vaccine-derived polioviruses. J. Infect. Dis. *210* (*Suppl 1*), S283–S293.

Burrill, C.P., Strings, V.R., and Andino, R. (2013). Poliovirus: generation, quantification, propagation, purification, and storage. Curr Protoc Microbiol. *29*, 15H–1.

Chao, L. (1990). Fitness of RNA virus decreased by Muller's ratchet. Nature *348*, 454–455.

Clark, M.F., Lister, R.M., and Bar-Joseph, M. (1986). ELISA techniques. Methods Enzymol. *118*, 742–766.

Contreras, G., Dimock, K., Furesz, J., Gardell, C., Hazlett, D., Karpinski, K., McCorkle, G., and Wu, L. (1992). Genetic characterization of Sabin types 1 and 3 poliovaccine virus following serial passage in the human intestinal tract. Biologicals *20*, 15–26.

Curigliano, G. (2018). Cancer Evolution as the New Frontier of Precision Medicine. Handb. Exp. Pharmacol. *249*, 289–297.

Davis, L.E., Bodian, D., Price, D., Butler, I.J., and Vickers, J.H. (1977). Chronic progressive poliomyelitis secondary to vaccination of an immunodeficient child. N. Engl. J. Med. *297*, 241–245.

Domingo, E., and Holland, J. (1994). Mutations and rapid evolution of RNA viruses (New York: Raven Press).

Domingo, E., Martin, V., Perales, C., Grande-Pérez, A., García-Arriaza, J., and Arias, A. (2006). Viruses as quasispecies: biological implications. Curr. Top. Microbiol. Immunol. *299*, 51–82.

Duarte, E., Clarke, D., Moya, A., Domingo, E., and Holland, J. (1992). Rapid fitness losses in mammalian RNA virus clones due to Muller's ratchet. Proc. Natl. Acad. Sci. USA *89*, 6015–6019.

Dunn, G., Begg, N.T., Cammack, N., and Minor, P.D. (1990). Virus excretion and mutation by infants following primary vaccination with live oral poliovaccine from two sources. J. Med. Virol. *32*, 92–95.

Evans, D.M., Dunn, G., Minor, P.D., Schild, G.C., Cann, A.J., Stanway, G., Almond, J.W., Currey, K., and Maizel, J.V., Jr. (1985). Increased neurovirulence associated with a single nucleotide change in a noncoding region of the Sabin type 3 poliovaccine genome. Nature *314*, 548–550.

Fisher, R.A. (1930). The Genetical Theory of Natural Selection (Oxford, UK: Oxford Univ. Press).

Goodfellow, I., Chaudhry, Y., Richardson, A., Meredith, J., Almond, J.W., Barclay, W., and Evans, D.J. (2000). Identification of a cis-acting replication element within the poliovirus coding region. J. Virol. *74*, 4590–4600.

Guest, S., Pilipenko, E., Sharma, K., Chumakov, K., and Roos, R.P. (2004). Molecular mechanisms of attenuation of the Sabin strain of poliovirus type 3. J. Virol. *78*, 11097–11107.

Holm, S. (2019). A simple sequentially rejective multiple test procedure. Scand Stat Theory Appl. *6*, 65–70.

Jorba, J., Diop, O.M., Iber, J., Henderson, E., Zhao, K., Sutter, R.W., Wassilak, S.G.F., and Burns, C.C. (2018). Update on Vaccine-Derived Polioviruses - Worldwide, January 2017-June 2018. MMWR Morb. Mortal. Wkly. Rep. *67*, 1189–1194.

Kauder, S.E., and Racaniello, V.R. (2004). Poliovirus tropism and attenuation are determined after internal ribosome entry. J. Clin. Invest. *113*, 1743–1753.

Kew, O.M., and Nottay, B.K. (1984). Molecular epidemiology of polioviruses. Rev. Infect. Dis. *6* (*Suppl 2*), S499–S504.

Kew, O., Morris-Glasgow, V., Landaverde, M., Burns, C., Shaw, J., Garib, Z., André, J., Blackman, E., Freeman, C.J., Jorba, J., et al. (2002). Outbreak of poliomyelitis in Hispaniola associated with circulating type 1 vaccine-derived poliovirus. Science *296*, 356–359.

Kew, O.M., Sutter, R.W., de Gourville, E.M., Dowdle, W.R., and Pallansch, M.A. (2005). Vaccine-derived polioviruses and the endgame strategy for global polio eradication. Annu. Rev. Microbiol. *59*, 587–635.

Knowlson, S., Burlison, J., Giles, E., Fox, H., Macadam, A.J., and Minor, P.D. (2015). New Strains Intended for the Production of Inactivated Polio Vaccine at Low-Containment After Eradication. PLoS Pathog. *11*, e1005316.

Konopka-Anstadt, Jennifer, Campagnoli, Ray, Vincent, Annelet, Shaw, Jing, Wei, Ling, Wynn, Nhien, Smithee, Shane, Bujaki, Erika, Yeh, Ming Te, Laassri, Majid, Zagorodnyaya, Tatiana, Weiner, Amy, Chumakov, Konstantin, Andino, Raul, Macadam, Andrew, Kew, Olen, and Burns, Cara (2020). Development of a new oral poliovirus vaccine for the eradication end game using codon deoptimization. NPJ Vaccines. https://doi.org/10.1038/s41541-020-0176-7.

Korotkova, E., Laassri, M., Zagorodnyaya, T., Petrovskaya, S., Rodionova, E., Cherkasova, E., Gmyl, A., Ivanova, O.E., Eremeeva, T.P., Lipskaya, G.Y., et al. (2017). Pressure for Pattern-Specific Intertypic Recombination between Sabin Polioviruses: Evolutionary Implications. Viruses *9*, 353.

Lauring, A.S., and Andino, R. (2010). Quasispecies theory and the behavior of RNA viruses. PLoS Pathog. *6*, e1001005.

Lauring, A.S., Jones, J.O., and Andino, R. (2010). Rationalizing the development of live attenuated virus vaccines. Nat. Biotechnol. *28*, 573–579.

Lenski, R.E., Mongold, J.A., Sniegowski, P.D., Travisano, M., Vasi, F., Gerrish, P.J., and Schmidt, T.M. (1998). Evolution of competitive fitness in experimental populations of E. coli: what makes one genotype a better competitor than another? Antonie van Leeuwenhoek *73*, 35–47.

Lowry, K., Woodman, A., Cook, J., and Evans, D.J. (2014). Recombination in enteroviruses is a biphasic replicative process involving the generation of greater-than genome length 'imprecise' intermediates. PLoS Pathog. *10*, e1004191.

Macadam, A.J., Ferguson, G., Burlison, J., Stone, D., Skuce, R., Almond, J.W., and Minor, P.D. (1992). Correlation of RNA secondary structure and attenuation of Sabin vaccine strains of poliovirus in tissue culture. Virology *189*, 415–422.

Macadam, A.J., Stone, D.M., Almond, J.W., and Minor, P.D. (1994). The 5′ noncoding region and virulence of poliovirus vaccine strains. Trends Microbiol. *2*, 449–454.

Macadam, A.J., Ferguson, G., Stone, D.M., Meredith, J., Knowlson, S., Auda, G., Almond, J.W., and Minor, P.D. (2006). Rational design of genetically stable, live-attenuated poliovirus vaccines of all three serotypes: relevance to poliomyelitis eradication. J. Virol. *80*, 8653–8663.

Manor, Y., Handsher, R., Halmut, T., Neuman, M., Bobrov, A., Rudich, H., Vonsover, A., Shulman, L., Kew, O., and Mendelson, E. (1999). Detection of poliovirus circulation by environmental surveillance in the absence of clinical cases in Israel and the Palestinian authority. J. Clin. Microbiol. *37*, 1670–1675.

McKnight, K.L., and Lemon, S.M. (1996). Capsid coding sequence is required for efficient replication of human rhinovirus 14 RNA. J. Virol. *70*, 1941–1952.

Minor, P.D. (2012). Polio vaccines and the eradication of poliomyelitis. Lancet *380*, 454–455.

Minor, P., Dunn, G., Almond, J.W., and MacAdam, A. (1993). Stability of attenuated poliovirus strain genotypes in the human intestinal tract. In Measles and Poliomyelitis (Springer Vienna), pp. 391–399.

Neverov, A., and Chumakov, K. (2010). Massively parallel sequencing for monitoring genetic consistency and quality control of live viral vaccines. Proc. Natl. Acad. Sci. USA *107*, 20063–20068.

Pallansch, M.A. (2018). Ending Use of Oral Poliovirus Vaccine - A Difficult Move in the Polio Endgame. N. Engl. J. Med. *379*, 801–803.

Pfeiffer, J.K., and Kirkegaard, K. (2003). A single mutation in poliovirus RNA-dependent RNA polymerase confers resistance to mutagenic nucleotide analogs via increased fidelity. Proc. Natl. Acad. Sci. USA *100*, 7289–7294.

Platt, L.R., Estívariz, C.F., and Sutter, R.W. (2014). Vaccine-associated paralytic poliomyelitis: a review of the epidemiology and estimation of the global burden. J. Infect. Dis. *210* (*Suppl 1*), S380–S389.

Polio Global Eradication Initiative (2020). This Week. http://polioeradication.org/polio-today/polio-now/this-week/.

Pollard, S.R., Dunn, G., Cammack, N., Minor, P.D., and Almond, J.W. (1989). Nucleotide sequence of a neurovirulent variant of the type 2 oral poliovirus vaccine. J. Virol. *63*, 4949–4951.

Reddy, B.Y., Miller, D.M., and Tsao, H. (2017). Somatic driver mutations in melanoma. Cancer *123* (S11), 2104–2117.

Roberts, L. (2018). Polio outbreaks in the DRC threaten eradication effort. Science *361*, 10–11.

Rouzine, I.M., Rodrigo, A., and Coffin, J.M. (2001). Transition between stochastic evolution and deterministic evolution in the presence of selection: general theory and application to virology. Microbiol. Mol. Biol. Rev. *65*, 151–185.

Rowe, A., Ferguson, G.L., Minor, P.D., and Macadam, A.J. (2000). Coding changes in the poliovirus protease 2A compensate for 5'NCR domain V disruptions in a cell-specific manner. Virology *269*, 284–293.

Roze, D., and Barton, N.H. (2006). The Hill-Robertson effect and the evolution of recombination. Genetics *173*, 1793–1811.

Stern, A., Yeh, M.T., Zinger, T., Smith, M., Wright, C., Ling, G., Nielsen, R., Macadam, A., and Andino, R. (2017). The Evolutionary Pathway to Virulence of an RNA Virus. Cell *169*, 35–46.e19.

Svitkin, Y.V., Cammack, N., Minor, P.D., and Almond, J.W. (1990). Translation deficiency of the Sabin type 3 poliovirus genome: association with an attenuating mutation C472——U. Virology *175*, 103–109.

Temko, D., Tomlinson, I., Severini, S., Schuster-Boeckler, B., and Graham, T. (2017). The effects of mutational process and selection on driver mutations across cancer types. Nat Commun. *9*, 1–10.

Thompson, K.M., and Kalkowska, D.A. (2019). Logistical challenges and assumptions for modeling the failure of global cessation of oral poliovirus vaccine (OPV). Expert Rev. Vaccines *18*, 725–736.

Van Damme, P., De Coster, I., Bandyopadhyay, A.S., Revets, H., Withanage, K., De Smedt, P., Suykens, L., Oberste, M.S., Weldon, W.C., Costa-Clemens, S.A., et al. (2019). The safety and immunogenicity of two novel live attenuated monovalent (serotype 2) oral poliovirus vaccines in healthy adults: a double-blind, single-centre phase 1 study. Lancet *394*, 148–158.

Vignuzzi, M., Stone, J.K., Arnold, J.J., Cameron, C.E., and Andino, R. (2006). Quasispecies diversity determines pathogenesis through cooperative interactions in a viral population. Nature *439*, 344–348.

Wilm, A., Aw, P.P., Bertrand, D., Yeo, G.H., Ong, S.H., Wong, C.H., Khor, C.C., Petric, R., Hibberd, M.L., and Nagarajan, N. (2012). LoFreq: a sequence-quality aware, ultra-sensitive variant caller for uncovering cell-population heterogeneity from high-throughput sequencing datasets. Nucleic Acids Res. *40*, 11189–11201.

Worobey, M., and Holmes, E.C. (1999). Evolutionary aspects of recombination in RNA viruses. J. Gen. Virol. *80*, 2535–2543.

Xiao, Y., Rouzine, I.M., Bianco, S., Acevedo, A., Goldstein, E.F., Farkov, M., Brodsky, L., and Andino, R. (2016). RNA Recombination Enhances Adaptability and Is Required for Virus Spread and Virulence. Cell Host Microbe *19*, 493–503.

Xiao, Y., Dolan, P.T., Goldstein, E.F., Li, M., Farkov, M., Brodsky, L., and Andino, R. (2017a). Poliovirus intrahost evolution is required to overcome tissue-specific innate immune responses. Nat. Commun. *8*, 375.

Xiao, Y., Rouzine, I.M., Bianco, S., Acevedo, A., Goldstein, E.F., Farkov, M., Brodsky, L., and Andino, R. (2017b). RNA Recombination Enhances Adaptability and Is Required for Virus Spread and Virulence. Cell Host Microbe *22*, 420.

World Health Organization. (2015). WHO SOP for neurovirulence testing of Oral Polio Vaccine using TgPVR21 transgenic mice. https://www.who.int/biologicals/vaccines/POLIO_SOP_TgmNVT_SOPv7_30_June2015_CLEAN2.pdf?ua=1.

STAR★METHODS

KEY RESOURCES TABLE

REAGENT or RESOURCE	SOURCE	IDENTIFIER
Bacterial and Virus Strains		
Oral Polio Vaccine Sabin Type 2 reference strain (OPV2)	NIBSC	01/530
Poliovirus Type 3 Leon	NIBSC	PV3 Leon
Biological Samples		
Immunized mouse sera	This study	N/A
Human serum samples	Van Damme et al., 2019	N/A
Human feces samples	Van Damme et al., 2019	N/A
Chemicals, Peptides, and Recombinant Proteins		
Bovine albumin fraction V, 7.5%	GIBCO	15260037
Formaldehyde solution	Sigma	252549
Crystal violet	Sigma	C0775
Methanol	Fisher Scientific	A412-500
Critical Commercial Assays		
KAPA Stranded mRNA-Seq Kit with KAPA mRNA Capture Beads	KAPA Biosystems	Cat# KK8420
HiSeq Rapid Cluster Kit v2	Illumina	Cat# GD-402-4002
HiSeq Rapid SBS Kit v2	Illumina	Cat# FC-402-4022
Miseq Reagent Kit v2 (300 cycle)	Illumina	Cat# MS-102-2002
ZR Viral RNA Kit	ZYMO Research	Cat# R1035
SuperScript III One-Step RT-PCR System with Platinum Taq High Fidelity DNA Polymerase	Invitrogen	Cat# 12574030
AMPure XP magnetic beads	Beckman Coulter	Cat# A63880
Qubit High Sensitivity dsDNA assay	Life Technologies	Cat# Q32854
Luciferase assay system	Promega	Cat# E1500
Phusion High-Fidelity DNA Polymerase	NEB	M0530S
Deposited Data		
In vitro accelerated evolution under higher temperature	This paper	SRA: PRJNA605034
Poliovirus shed from OPV vaccinees	This paper	SRA: PRJNA605034
Variant calls for deep sequencing	This paper	Mendeley data https://doi.org/10.17632/3n5ztwx66m.1
Experimental Models: Cell Lines		
Human: HeLa S3 cells	ATCC	CCL2.2
Human: Hep 2C cells	NIBSC	740502
Monkey: Vero cells	ATCC	CCL81
Mouse: L20B cells	NIBSC	081102
Experimental Models: Organisms/Strains		
Mouse: PVRTg21-IFNR-ko	Gift from Satoshi Koike, Tokyo Metropolitan Institute of Medical Science	Tg21/IFNR-ko
Mouse: PVRTg66	NIBSC	PVRTg66
Oligonucleotides		
NEXTFlex RNA-Seq Barcodes	BIOO Scientific	#512914

(*Continued on next page*)

Continued

REAGENT or RESOURCE	SOURCE	IDENTIFIER
Primer D53N-F (5′-GCTCAGGACAAATTTTG AAGAAGCAATATTCTC-3′)	This paper	D53N-F
Primer D53N-R (5′-CTTCAAA ATTTGTCCTGAGCCTGGGAT CATTTTTG-3′)	This paper	D53N-R
Primer K38RF (5′- GAAGGGGTGAGAGA ACCAGCAGTCCTTACCAA-3′)	This paper	K38R-F
Primer K38RR (5′- CTGCTGGTTCTCTCAC CCCTTCAAACACATAGTG-3′)	This paper	K38R-R
Primer P234SF (5′- CATCACTCAGCTCAGCC TGGTTTGAGGCACTCAA-3′)	This paper	P243S-F
Primer P234RR (5′- CAAACCAGGCTGAGCT GAGTGATGCATCATAACC-3′)	This paper	P243S-R
Primer L445MF (5′- GGGAGAGCTGTGTTGCT CCCAGAGTACTCTACATTGTAC-3′)	This paper	L445M-F
Primer L445MR (5′- CTGGGAGCAACACAG CTCTCCCAATTGGCACACTCCTG-3′)	This paper	L445M-R
Primer S2-d3D-F (5′- GGATGAAAC CATGATAATAAGTGGGATACCCG ATCATAAATGCTCC-3′)	This paper	S2-d3D-F
Primer S2-d3D-R (5′- GGGTATCC CACTTATTATCATGGTTTCATCC ATTGAATCTCACCTTG-3′)	This paper	S2-d3D-R
Primer F-S2REP-5UTR-EcoRI (5′- CAT GATTACGAATTCGAGCTCTAATAC-3′)	This paper	F-S2REP-5UTR-EcoRI
Primer R-S2REP-5UTR-SalI (5′- CTTCC ATGTCGACAACTTTCTGTGATGAA ACTTGGGCGC-3′)	This paper	R-S2REP-5UTR-SalI
Primer F-S2REP-FLuc-SalI (5′- GAAA GTTGTCGACATGGAAGACGCCA AAAACATAAAG-3′)	This paper	F-S2REP-FLuc-SalI
Primer R-S2REP-FLuc-EagI (5′- CTAGT GGCGGCCGCACGGCGATCTTTCCG CCCTTCTTGG-3′)	This paper	R-S2REP-FLuc-EagI
Primer F-S2REP-NSR-EagI (5′- GCCG TGCGGCCGCCACTAGAAAAG GGATTAACGAC-3′)	This paper	F-S2REP-NSR-EagI
Primer R-S2REP-NSR-SbfI (5′- GGTAAC GCCAGGGTTTTCCCAGTCACGACG-3′)	This paper	R-S2REP-NSR-SbfI
Recombinant DNA		
Plasmid: pRA-Sabin2	This paper	pRA-Sabin2
Plasmid: pRA-Sabin2-D53N	This paper	pRA-Sabin2-D53N
Plasmid: pRA-Sabin2-K38R	This paper	pRA-Sabin2-K38R
Plasmid: pRA-Sabin2-D53N/K38R	This paper	pRA-Sabin2-53/38
Plasmid: pS2/S15domV/cre5	This paper	pRA-Sabin2-2909
Plasmid: pS2/S15domV/cre5-D53N	This paper	pRA-Sabin2-398/481/2909
Plasmid: pS2/S15domV/cre5-K38R	This paper	pS2/S15domV/cre5-K38R
Plasmid: pS2/S15domV/cre5-D53N/K38R	This paper	pS2/S15domV/cre5-D53N/K38R
Plasmid: pSabin2-d3D	This paper	pSabin2-d3D
Plasmid: pSabin2-dCre	This paper	pSabin2-dCre
Plasmid: Sabin2 replicon	This paper	pS2Rep
Plasmid: pS2Rep-D53N	This paper	pS2Rep-D53N

(*Continued on next page*)

Continued

REAGENT or RESOURCE	SOURCE	IDENTIFIER
Plasmid: pS2Rep-K38R	This paper	pS2Rep-K38R
Software and Algorithms		
Prism	GraphPad Software	https://www.graphpad.com
LoFreq	Wilm et al., 2012	https://csb5.github.io/lofreq/
Python	Python Software Foundation	https://www.python.org
Chimera	UCSF	https://www.cgl.ucsf.edu/chimera/

RESOURCE AVAILABILITY

Lead Contact

Further information and requests for resources and reagents should be directed to and will be fulfilled by the Lead Contact, Raul Andino (Raul.Andino@ucsf.edu).

Materials Availability

All unique/stable reagents generated in this study are available from the Correspondence (Andrew.Macadam@nibsc.org; Raul.Andino@ucsf.edu) with a completed Materials Transfer Agreement.

Data and Code Availability

Sequencing data can be access on the SRA database, accession numbers PRJNA605034. Variant calls for sequencing can be accessed on the Mendeley data, https://doi.org/10.17632/3n5ztwx66m.1.

EXPERIMENTAL MODEL AND SUBJECT DETAILS

Cells and Viruses

HEp2C (NIBSC 740502), HeLa S3 (ATCC® CCL-2.2), Vero (ATCC® CCL-81), L20B (NIBSC 081102) cells were used in this study. Vero and L20B cells were maintained in Eagle's Minimum Essential Medium supplemented with 10% fetal bovine serum (FBS, Sigma-Aldrich) and 1X penicillin/streptomycin (Invitrogen) at 37°C with 5% CO_2. HEp2C and HeLa S3 cells were maintained as described (Burrill et al., 2013). Sabin SO+2/II master seed and human poliovirus 3 strain Leon were obtained from National Institute for Biological Standard and Controls (NIBSC, UK). Viruses were propagated and titered in HEp2C or HeLa S3 cells at 33°C or 37°C by standard plaque-forming assay and $TCID_{50}$.

Mouse Model

Mouse strain, PVRTg21 with type I interferon receptor knock-out (PVRTg21/IFNR-ko), was provided by Dr. Satoshi Koike, and maintained in a specific pathogen-free, AAALAC-certified animal facility at UCSF under and a 12 h light/dark cycle with standard chow diet provided. Four weeks old, sex-matched mice were used for immunogenicity and 10 days old for virulence test via i.p. infection. All procedures were performed in accordance with the guidelines of the Laboratory Animal Center of National Institutes of Health. The Institutional Animal Care and Use Committee of the University of California, San Francisco approved all animal protocols (Approved protocol No. AN128674-01A).

PVRTg66 mice of 6-8-week-old in groups of eight (weight and sex-matched) were used for i.s. neurovirulence test. Mouse experiments at NIBSC were performed under licenses PPL 80/2478 and PPL 70/8979 granted by the UK Home Office under the Animal (Scientific Procedures) Act 1986 revised 2013 and reviewed by the internal NIBSC Animal Welfare and Ethics Review Board before submission.

Human Subjects

The double-blind phase 1 trial was conducted at the University of Antwerp (Antwerp, Belgium) as described in more detail separately (Van Damme et al., 2019). Briefly, volunteers were recruited through local advertisement and screened with medical examination, laboratory testing, and also evaluated by two psychologists for mental fitness. Volunteers were excluded if they were previously vaccinated with OPV, any polio vaccine within 12 months, or any other vaccine within 28 days of the start of the study, or had any condition that the investigators believed would compromise participants' health. Eligible volunteers were healthy men or women (aged 21-50 years) with IPV-only polio vaccination histories. Baseline blood and stool samples were collected at the screening visit, and the first three volunteers were enrolled in the first group subsequently. Fifteen volunteers were enrolled in each group and the first participant decided the vaccine strain to be received for that group by opening one of two envelops. The investigators considered the sample size of 15 per group reasonable and sufficient to evaluate the tested vaccine in a phase 1 trial, and was agreed by The Global Polio Eradication Initiative. The two groups were constituted with 13 males and 2 females with an average age of 31.1 ± 7.7 years

(mean with SD), and 12 males and 3 females of 33.5 ± 10.9 years old, respectively. All participants provided written consent at enrolment. The study was conducted in accordance with Declaration of Helsinki and ICH Good Clinical Practice guidelines and was approved by the university and hospital institutional review boards. The US Centers for Disease Control and Prevention's Human Research Protection Office reviewed the study protocol. An independent Data and Safety Monitoring Board oversaw the study.

METHOD DETAILS

Relocation and Optimization *of cre*

Recombination between species C enteroviruses that circulate in humans is very frequent (Kew and Nottay, 1984). Therefore, in the long-term, the stabilized attenuating S15*domV* region could be exchanged with *domV* derived from viruses co-circulating *in vivo*. Several reports have documented that exchange in Sabin2-derived strains (Kew and Nottay, 1984; Stern et al., 2017). To reduce the probability of loss of genetically stable, engineered S15*domV* by recombination, we relocated the essential *cre* element from its location in the 2C gene to a 5′ noncoding region location, upstream of *domV*. Removal of *domV* would then require two recombination events in the same genome, on either side of *domV*, since a single event would also remove the *cre*, resulting in loss of viability.

Constructs were made with a disabled *cre* element (2C *cre* mutated) as described (Goodfellow et al., 2000) with and without a relocated *cre* (5′*cre*)(Figure S2A). The 2C *cre* mutated virus was non-viable, and blind passaging experiments confirmed that this alteration of 2C *cre* cannot revert to a functional structure (not shown); insertion of a new *cre*, identical to that in Sabin2 2C, between nucleotides 120 and 121 restored viability. However, this initial construct was significantly less fit as determined by the one-step growth curve (not shown) and plaque assay (Figure S3A), more temperature-sensitive in Vero cells (Figure S2B), and more attenuated than Sabin2 after intraspinal inoculation (Figure S2C). Selecting fitter variants in cell culture and analyzing sequence changes suggested the strength of the stem structure needed to be increased, possibly so that the functional structure was also the lowest energy fold after relocation within the 5′-UTR. A number of constructs were made and recovered, iteratively improving the design each time (Figure S2A) until a suitable construct (*cre*5) was produced that incorporated several features: a more favorable loop sequence, two strengthened base-pairs (U-G→C-G), a longer stem by four base-pairs, and two UAG (STOP) triplets which would be in-frame if the *cre* was somehow recombined into the original 2C location (Figure S2A).

Initial *cre* mutants of Sabin2 and S2/S15*domV* were significantly more attenuated than Sabin2 as were *cre*2, *cre*3, and *cre4* mutants (Figure S2C). This was not unexpected as the viruses were also more temperature-sensitive than Sabin2 (Figure S2B). The *cre*5 mutant, despite showing similar temperature-sensitivity to Sabin, was also more attenuated in the TgPVR model. This suggested that *cre* relocation itself has an attenuating effect, at least in mice.

One-step growth yields of *cre* mutants in Vero cells were lower for all mutants except S2/*cre*5/S15*domV* (Figure S3B). All mutants except S2/*cre*5/S15*domV* displaced a more pronounced temperature-sensitivity phenotype in Vero cells than Sabin2 (Figures S2B and S2C). Therefore, since S2/*cre*5/S15*domV* was similar to Sabin2, this virus was chosen to take forward to the next stage.

Selection of *HiFi* Mutations

High-fidelity Sabin2 variants were selected in the presence of mutagens as described (Pfeiffer and Kirkegaard, 2003; Vignuzzi et al., 2006). Briefly, HeLa S3 cells were seeded in 10-cm dishes (10^7 cells per dish) and cultured at 37°C the day before the experiment. The cells were pretreated with 100 μM of ribavirin or mock (culture media) for 4 h at 37°C before virus infection. Culture medium was then removed, and 10^6 pfu of Sabin2 was added to the pretreated cells. The cells were left at room temperature for 1 h to allow virus adsorption, washed once with PBS, added with culture medium with/without 100 μM of ribavirin, and kept at 33°C for 10 h until harvest (Figure 2A). Virus titers were determined with standard plaque-forming assay for the next passage. Eight passages were performed to generate virus populations of directed evolution (**Fig. SA**). Highly accurate next-generation sequencing (CirSeq) analysis was performed to identify candidate determinants of replication fidelity as described (Acevedo et al., 2014). Eleven mutations selected under mutagenic conditions (ribavirin) were identified and introduced in the Sabin2 infectious clone to confirm ribavirin resistance.

Ribavirin Resistance Assay

HeLa S3 cells were pre-treated with ribavirin (0, 100, 200, 400 or 800 μM) at 37°C for 4 h. Sabin2-*HiFi* mutant viruses were added to cells at m.o.i. of 0.01 in triplicates and let stand at room temperature for 1 h. The cells were washed once with PBS, added with viral medium (containing 0, 100, 200, 400 or 800 μM of ribavirin) and incubated at 33°C for 24 h. Virus titer was determined with a standard $TCID_{50}$ assay.

Construction of Sabin2-eGFP Virus Infectious Clone

PCR amplification was performed to generate DNA fragments of 5′-UTR, P1, and P2P3 region and eGFP with primers carrying restriction enzyme sites. Each DNA fragment was gel-purified, digested with restriction enzymes and ligated to the pre-digested vector. Virus generation from infectious clone and quantification with TCID50 have been described (Burrill et al., 2013; Xiao et al., 2016).

Selection of Recombination Deficient Virus

To select potential recombination determinant, HeLa S3 cells were seeded onto 96-well plates (10^4 cells per well) and cultured at 37°C with 5% CO_2 for 24 h, and then infected with 100 μL of Sabin2-eGFP (adjusted to 2.5 $TCID_{50}$/mL). The plates were kept in a

33°C incubator for seven days. One row of wells on each 96-well plate was left uninfected as a negative control. The ratio of the number of GFP-positive wells (determined by ELISA plate reader) to CPE-positive wells (determined visually under a microscope) was monitored to evaluate GFP retention in virus populations. Viruses in GFP-positive wells were harvested, quantified by $TCID_{50}$ and used for subsequent passages. When GFP retention percentage reached 90%, viruses from GFP-positive wells were sequenced to identify candidate recombination defective determinants (five candidates were identified).

Generation of Sabin2-*HiFi* and Sabin2-*Rec1* Viruses

The strategy for the construction of infectious clones carrying candidate *HiFi* and *Rec1* mutations has been described (Burrill et al., 2013; Xiao et al., 2016). Virus generation from infectious clone and plaque-forming assay have been described (Burrill et al., 2013; Xiao et al., 2016).

Construction of Sabin2 Replicon and 3D/CRE Truncation

Construction of Sabin2 replicon, S2Rep, was very similar to that of Sabin2-eGFP. DNA fragments of 5′-UTR, P2P3 region of Sabin2, and firefly luciferase gene (pGL3, Promega) were PCR amplified with primers carrying restriction enzyme sites, gel-purified, digested and ligated to a pre-digested vector. To construct a Sabin2 infectious clone with truncated CRE (S2dCre) or 3D (S2d3D), PCR was performed with primers carrying mutations in CRE and stop codons for the 38th–40th amino acids of 3D RNA polymerase, digested with *Dpn I* and transformed into SURE 2 cells (Agilent). Clones with introduced mutations were verified by Sanger sequencing and used as templates for subsequent mutagenesis. *Rec* mutations were introduced into S2dCre with primers carrying mutations as described above.

Recombination Validation Assay

In vitro transcribed RNA from S2dCre, S2dCre-*Rec* and S2d3D (500 ng each per well in a 6-well plate) were co-transfected into 60% confluent L929 cells in triplicates with lipofectamine 3000 (Invitrogen), following manufacturer's instruction. Plates were kept in 33°C incubator and harvested 48 h post-transfection. Cells were frozen and thawed three times and cleared at 3,000 rpm for 10 min under 4°C. The recombinant virus is tittered with plaque-forming assay (Figure 2F).

Replicon Replication Assay

In vitro transcribed S2Rep, S2Rep-*HiFi*, and S2Rep-*Rec1* RNAs were electroporated into HeLa S3 cells in triplicates as described above. Electroporated cells were plated onto 12-well, incubated in 33°C incubator, and harvested at 0, 2, 4, 6, 8, 10, 12 and 24 h after electroporation. Upon harvest, cells were washed once with PBS and lysed in 1X PLB (Passive Lysis Buffer, Promega) for luciferase assay. For h-0 samples, cells were centrifuged briefly at 180 *xg* for 5 min and resuspended in 1X PLB. Firefly luciferase activity was determined with the Luciferase Assay System (Promega), following the manufacturer's protocol. Results are shown as mean with SD (Figure 2H).

Construction of nOPV Variants

Design and construction of the strains were carried out in two phases: an initial phase where individual strategies were tested on their own and then a second phase where results of the characterizations of these viruses are fed back into the design process.

Sabin2 Infectious Clone

Construction of the Sabin2 infectious clone from Sabin SO+2/II master seed has been described (Pollard et al., 1989).

nOPV2(S2/cre5/S15domV/Rec1/HiFi3)

The Sabin2 infectious clone was used as a template to construct nOPV2 (cre and S15*domV*) and other *domV* or 5′*cre* variants. Nucleotide changes were introduced by site-directed mutagenesis or by *de novo* synthesis of DNA fragments (the introduced changes are shown in Figure 1). *HiFi* and *Rec1* mutations were introduced into 3D RNA polymerase of S2/Cre5/S15*domV* by site-directed mutagenesis. Briefly, PCR was performed with primers carrying the changed codons. The PCR products were digested with *Dpn I* (NEB) and transformed into SURE2 (Agilent) competent cells. Primer sequences: 5′-GCTCAGGACAAATTTTGAAGAAGCAATATTCTC-3′ and 5′-CTTCAAAATTTGTCCTGAGCCTGGGATCATTTTTG-3′ (forward and reverse for *HiFi*); 5′-GAAGGGGTGAGAGAACCA GCAGTCCTTACCAA-3′ and 5′-CTGCTGGTTCTCTCACCCCTTCAAACACATAGTG-3′ (forward and reverse for *Rec1*). Clones were verified with Sanger sequencing and used for virus recovery.

Virus Recovery

Protocols for virus recovery, including *in vitro* transcription, RNA electroporation, harvest, virus propagation, and quantification with standard plaque-forming assay were described (Burrill et al., 2013). Viruses recovered from electroporated cells (P0) were amplified as described (Burrill et al., 2013) to generate P1 viruses used in experiments.

Virus Growth Analysis

One-step growth analysis was performed as described (Burrill et al., 2013) except the infected cells were incubated at 33°C. Virus yield was evaluated in triplicates with Vero cells pre-seeded onto 6-well plates (10^6 cells per well) the day before the experiment. Briefly, viruses were added to infect cells at an MOI of 0.01 in triplicates and let stand at room temperature for 1 h to allow virus

adsorption. After one wash with PBS, viral medium was added to each well, and the infected cells were incubated at 33°C, 35°C, 36°C, 37°C or 39°C for 48 h. Viruses were harvested by three freeze-thaw cycles, and cell debris was removed by centrifugation. Plaque forming assay was performed to determine virus titer.

Virus Passages under Accelerated Evolution

To study virus evolution in cell culture, serial virus passages were performed with 10-cm dishes pre-seeded with 10^7 Vero cells. Sabin2 and nOPV2 were added to cells at an MOI of 0.1 and left at room temperature for 1 h to allow virus adsorption. Viral inoculum was then refreshed with viral medium, and infected cells were incubated at 37°C for 10 h. Viruses were harvested and quantified as described above and used for subsequent passages. Ten passages were performed to prepare virus populations for genetic stability assay.

Next-Generation Sequencing

Virus Population from Accelerated Evolution

Viral RNA was isolated from the viral population of the 10th passage of accelerated evolution or from stool samples obtained from vaccines (Van Damme et al., 2019). RNA was isolated from cell-culture samples using the ZR Viral RNA kit (ZYMO Research), according to the manufacturer's protocol. KAPA Stranded mRNA-Seq kit (KAPA Biosystems) was used to prepare the RNA libraries for sequencing. Briefly, 1 μg of RNA samples was further purified twice by using KAPA mRNA Capture Beads and eluted into 11 μl of RNase-free water. The purified RNA was mixed with an equal volume of KAPA Fragment Buffer, incubated at 94°C to generate fragments of 200–300 nucleotides. Next, cDNA was synthesized using KAPA 1st Strand Synthesis Mater Mix and random primers. cDNA was further converted into double-stranded cDNA with KAPA 2nd Strand Synthesis and Marking Mater Mix, followed by a purification with AMPure reagent. The double-stranded cDNA was treated with KAPA A-Tailing Master and ligated to NEXTflex RNA-Seq Barcodes (BIOO SCIENTIFIC). The libraries were cleaned up with 1X SPRI cleanup procedure twice and then enriched using 10 cycles of PCR and cleanup with AMPure Reagent. The library fragment size distribution was determined by using Bioanalyzer High Sensitivity DNA Assay (Agilent), and concentrations determined by using the KAPA Library Quantification Kit for Illumina Platform (KAPA Biosystems). Deep sequencing was performed using MiSeq (Illumina) producing 300-nucleotide paired-end reads. Sequencing reads were analyzed by using Lofreq to determine variant frequency (Wilm et al., 2012).

Shed Virus from Vaccinees in Clinical Trial

The exploratory endpoint specimen (EES) was defined as the last stool sample provided by each subject with adequate levels of virus ($\geq$ 4 log_{10} $CCID_{50}$ per gram) for deep sequencing assays. Detected polioviruses in EES samples were isolated and amplified on HEp2c cells for 3 days at 33°C with a fixed number of passages to achieve the required quantity of virus for neurovirulence. Deep sequencing was performed on both cell-culture-amplified virus, and viral RNA was isolated from the EES of each subject to assess the retention of key genetic modifications. Viral RNA was isolated from amplified virus stock or stool using a QiaAmp Viral RNA mini kit (QIAGEN), followed by cDNA synthesis and full-length poliovirus genome amplification (KOD Xtreme Hot Start DNA polymerase kit, Millipore). Fragmentation and library preparation by Nextera XT (Illumina) were followed by 300-cycle paired-end sequencing using MiSeq reagent kit v3 reagents on a MiSeq sequencer with MiSeq analysis software version 1.8.46 (all available from Illumina) to generate FASTQ files.

Diversity (D_i) in the sequenced populations was summarized as the sum across individuals of Shannon's entropy values for individual sequence windows along the sequence, calculated as:

$$D_w = -\sum_{j}^{l}\sum_{i}^{n} p_{ij}\left(\log\left(p_{ij}\right)\right)$$

where, n is the number of variants in window, w, and l is the number of subjects. A summary of these analyses is presented in Figure 7A.

All sequencing reads from both *in vivo* and *in vitro* studies have been deposited in NCBI short-read archive under project number PRJNA605034. The variant calls from these sequencing studies have been deposited at Mendeley data, https://doi.org/10.17632/3n5ztwx66m.1.

Neurovirulence Test in Tg66 Mice (TgmNVT)

The potential neurovirulence of nOPV2 was assessed in transgenic mice expressing the human poliovirus receptor (TgPVR mice). Intraspinal inoculation of Tg PVR mice was performed using a method adapted from the standard operating procedure (SOP) "WHO neurovirulence test of type 2 live poliomyelitis vaccines (oral) in transgenic mice susceptible to poliovirus" available from the WHO; the adaptation of the SOP included using higher doses and fewer mice per dose (WHO SOP). Additionally, Tg66 mice (which also express the human poliovirus receptor) (Knowlson et al., 2015) were used in substitution for the TgPVR21 strain used in the WHO assay. Both strains have similar sensitivities to Sabin2 when inoculated by the intraspinal route. Briefly, 6–8-week-old mice in groups of eight (weight and sex-matched) were sedated and inoculated into the lumbar region of the spinal cord with 5 μl of each dose and observed for occurrence of paralysis for up to 14 days. Mice with paresis/paralysis were scored positive, and mice surviving for 14 days with no clinical signs were scored negative. When possible, a paralyzing dose for 50% of mice (PD_{50}) was calculated using the Spearman-Karber method.

Recombination Assay

Neurovirulent Sabin2 revertant isolates either have an A-G mutation nt 481 in *domV* or they are recombinants containing wild-type *domV* sequences from other enteroviruses. A cell culture assay was designed to determine if *cre* relocation to a position in the 5′-UTR of PV2 upstream of *domV* inhibits the exchange of the *domV* S15 by recombination. Briefly, HEp2c cells were infected with nOPV variants and a potential donor of a wild-type *domV* under conditions permissive to both viruses (Figures 5A and S6A). The partner, wild-type 3 Leon (PV3), was a close relative of a different serotype to maximize the chances of recombination but allow selection with antibodies. In addition to the vaccine candidate precursor, which has the stabilized *domV* S15 and the relocated *cre*, two controls were used. One was Sabin2 and the other Sabin2 with a stabilized *domV* S15. Hep2c cells and culture conditions allowed replication of both viruses with minimal selection; the MOIs ensured the majority of cells were co-infected and that there was only a single round of replication.

HEp2c cells in 25-cm flasks were co-infected with one of three type 2 viruses and the wild-type 3 virus Leon at an MOI = 10 for type 2 and an MOI = 1 for type 3. Type 2 viruses were Sabin2, S2/S15*domV* and S2/cre5/S15*domV* (Figures 5A and 5B). Infected cells were incubated at 35°C until 100% CPE was observed. Flasks were frozen at −20°C and thawed, contents were centrifuged at 3,000 rpm for 10 min, and supernatants were removed and labeled P1. Subsequent passages in HEp2c cells were carried out in the same way with 1/100 dilutions of the harvest of the previous passage (labeled P2–P5).

After each passage, type 2 viruses were selected by neutralization of a 1/100 dilution of the HEp2c harvest with high-titer polyclonal anti-type 3 serum, infection of L20B cells and incubation at 37°C until 100% CPE was observed, conditions known to select reversion in *domV* of Sabin2 (see below) (Macadam et al., 2006). Supernatants were prepared as above and labeled “L1.” Subsequent passages in L20B cells were carried out in the same way with 1/100 dilutions of the harvest of the previous L20B passage (labeled L2). For example, a sample labeled “P3L2” was generated by three co-infections of HEp2c cells followed by 2 selection cycles in L20B cells.

The harvests of these cultures, using the *cre4* variant (Figure S6), were analyzed by PCR; the 5′-UTR sequences of type 2 viruses were amplified using a broad-specificity + sense primer near the very 5′ end and a Sabin2 capsid specific – sense primer (Figure S6B). Products were obtained for each successive Hep2c/L20B passage for each of the virus pairs. The product for the *cre* mutant is larger by 61 nt, which is the size of the inserted *cre5* (Figure 5B). Sanger sequencing of these products identified the predominant species in each mixture and revealed reversion and/or recombination frequency when it reaches 10% or more. The Sabin2 passage selected predominantly for 5′-UTR revertants: viruses with 481A-G. No recombination was apparent by this analysis. S15*domV* did not revert, consistent with published data on the genetic stability of S15 (Macadam et al., 2006), but sequences showed gradual enrichment of a product with a Leon sequence. The S2/cre4/S15*domV* mutant was stable throughout.

To identify recombinants specifically, we used PCR to amplify type 2 viruses with Leon 5′UTRs; the + sense primer was specific for Leon 5′UTR and – sense primer for Sabin2 capsid. Results for S15*domV* mirrored sequencing results. With Sabin2, it was clear that recombination and reversion had taken place, but the reversion frequency was higher than recombination frequency. Gradual enrichment of the recombinant suggested the recombinant was fitter than the revertant. Selection of Sabin2 revertants and S2/S15*domV* recombinants occurred at similar rates both for the first L20B passage (L1) and the second (L2) (not shown). No recombinants were detected in the co-culture of S2/cre4/S15*domV* and Leon. Therefore, the key attenuating determinants of this virus were genetically stable in terms of mutation and recombination.

In a second experiment, conducted the same way but using S2/cre5/S15*domV*, the progeny viruses were analyzed by deep-sequencing after whole-genome amplification. Reads were mapped to both type 2 and type 3 reference sequences (Figure 5B).

Immunogenicity Assay

Forty 4-week-old PVRTg21/IFNR-KO mice of were inoculated with 100 μl of inoculum that carried various doses (10^7 to 10^4 pfu of virus per mouse, 10 mice per dose) of Sabin2 or nOPV2, and another five mice were injected with 10^7 pfu of UVI-Sabin2 via i.p. route. Blood samples were collected from retro-orbital sinus at day 21 post-inoculation for a neutralization assay. To determine antibody titer, forty microliters of serum sample were added to 120 μl of diluent (DMEM, 1% BSA, 1X Penicillin/Streptomycin) and inactivated at 55°C for 30 min. A series of twofold serial dilutions of the inactivated sera was prepared with the same diluent on 96-well plates. Virus (100 $CCID_{50}$) was added to the diluted sera and left at 37°C for 2 h. The virus-serum mixture was then transferred to another 96-well plated pre-seeded with HEp2C or HeLa S3 cells (10^4 cells per well) and incubated in 33°C incubator for 7 days. The plates were fixed with formaldehyde and stained with 0.5% crystal violet to read CPE. The antibody titer was defined as the highest dilution sufficient to inhibit the development of CPE. A pre-immunized human serum was included as a neutralizing standard serum. Serum samples that showed no inhibition was assigned a titer of 4 to calculate mean titer and to prepare the graph.

Antigenicity Assay

A non-competitive sandwich ELISA assay (Clark et al., 1986)was used to measure reactivity with MAbs specific for four different antigenic sites present on native virus particles. Briefly, twofold dilutions of antigen were captured with a serotype-specific polyclonal antibody, then detected using serotype-specific monoclonal antibodies, followed by anti-mouse peroxidase conjugate. The reactivity of each test sample was evaluated against a Sabin2 reference.

QUANTIFICATION AND STATISTICAL ANALYSES

Data panels below were prepared and analyzed with Prism 8 (GraphPad) with statistical methods described below. Statistical significance was set as $p < 0.05$. All multiple test correction was performed using the Holm-Sidak Method (Holm, 2019), and reported below as "corrected *p*."

Figure 2. (C) IC_{50} and 95% confidence interval were estimated by nonlinear regression based on data from Figure S4. Bars show 95% confidence interval. (G) One-tailed Student's t test was performed to test if Rec mutations reduce recombination as compared with Sabin2 and *p* values of 0.0322, 0.2277, and 0.0313 were obtained for K38R, P243S, and L445M, respectively. (H) Unpaired Student's t test was performed to compare virus titers at collected time points and determined a significant difference with corrected *p* value of 0.0187 at 4 h post-infection between Sabin2 and *HiFi* (D53N).

Figure 3. (B) Data are shown as mean ± SD (standard derivation) of triplicates. Student's t tests were used to compare samples at each time, with correction for multiple comparisons. This analysis suggested significant differences in virus titer at 6 and 12 h (corrected p = 0.0080 and 0.0197, respectively) post-infection. (C) Student's t tests were used to compare virus yield of Sabin2 and nOPV2 at tested temperatures. No significant difference in virus titers were identified (Corrected p = 0.0538, 0.2991, 0.8744, 0.2711, 0.0786 for 33, 35, 36, 37 and 39°C, respectively).

Figure 6B. Data are presented as box and whisker plots with all data shown. Mann-Whitney test was performed to compare antibody titers induced by Sabin2 and nOPV2. The differences of antibody titers between Sabin2 and nOPV2 at the tested doses did not reach statistical significance (p = 0.5370, 0.1564, 0.0775, 0.0731 for 10^7, 10^6, 10^5 and 10^4, respectively).

Figure S3. (B) Data are shown as mean ± SD of triplicates. Unpaired, Student's *t*- tests were performed to compare virus titers at all time points and showed significant differences in virus titer at 3 and 6 h (corrected p = 0.8796, 0.0238, and 0.0136, 0.3419, 0.8796, and 0.5938 for 0, 3, 6, 9, 12, and 24-h post-infection, respectively). (C) Student's t tests were performed to compare virus titers of Sabin2 and S2/S15/*domV*/cre5 at tested temperatures and found significant differences for virus titers of at 33°C (corrected p = 0.0359, 0.3061, 0.6433, 0.3165, and 0.4295 for 33, 35, 36, 37 and 39°C, respectively).

Figure S4. Data are presented as mean ± standard error of mean (SEM). Unpaired, two-tailed, Student's t tests were performed and corrected *p* values of 0.9990, 0.0267, 0.1237, 0.0054 and 0.0065 were obtained for ribavirin concentrations at 0, 100, 200, 400 and 800 μM, respectively.

Figure S5A. Data are shown as mean ± SD of triplicates. Unpaired Student's t tests were used to compare differences in virus titers between S2/S15*domV*/cre5 and S2/S15*domV*/cre5/K38R/D53N, S2/S15*domV*/cre5/D53N, or S2/S15*domV*/cre5/K38R. Significant difference in virus titer was found only at 3 h post-infection (corrected p = 0.0017) between S2/S15domV/*cre5* and S2/S15domV/*cre5*/K38R.

Cell Host & Microbe, Volume *27*

Supplemental Information

Engineering the Live-Attenuated Polio Vaccine to Prevent Reversion to Virulence

Ming Te Yeh, Erika Bujaki, Patrick T. Dolan, Matthew Smith, Rahnuma Wahid, John Konz, Amy J. Weiner, Ananda S. Bandyopadhyay, Pierre Van Damme, Ilse De Coster, Hilde Revets, Andrew Macadam, and Raul Andino

Engineering the Live-Attenuated Polio Vaccine to Improve Safety by Preventing Reversion to Virulence

Yeh et al.

Supplementary figures

Figure S1

A

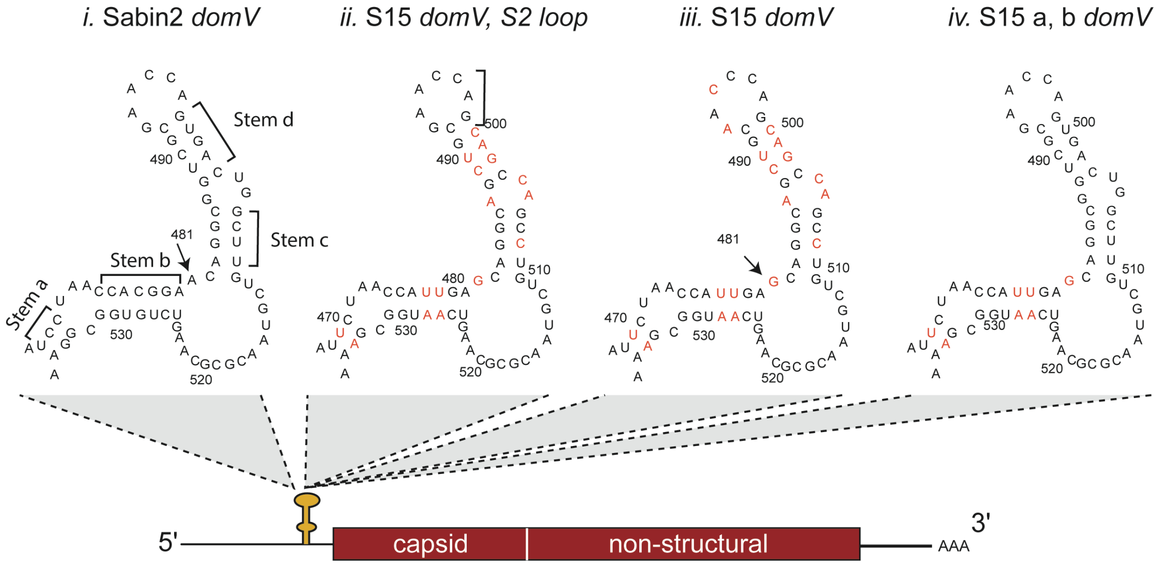

B

Temperature sensitivity

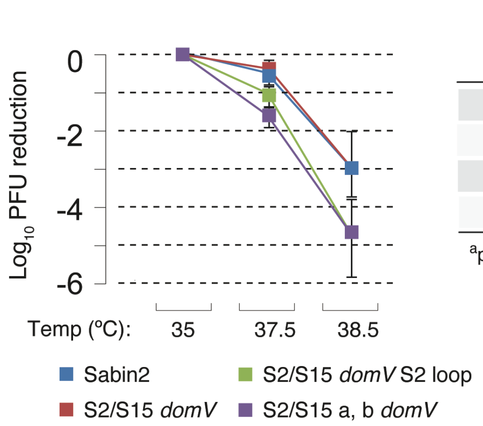

C

Neurovirulence

	Tg66-CBA, i.s. PD$_{50}$ ($log_{10}CCID_{50}$)
Sabin2	5.9
S2/S15 a, b *domV*	> 7.0 (0/8[a])
S2/S15 *domV* S2 loop	> 7.0 (0/8[a])
S2/S15 *domV*	6.0

[a]paralyzed/total at the highest dose

Figure S1. Related to Figure 1. Modifications within *domV*.

(A) Sabin2 domain V (*domV*) predicted secondary structures and optimized S15. The 5′-UTR *domV* is an RNA stem-loop structure involving nucleotides 468–535. Nucleotide

481A, the major attenuating mutation in Sabin2, is identified by arrow. Nucleotide differences between Sabin2 and modified *domV* are shown in red. *DomV* of Sabin2 was engineered to have all but the top-loop (S15 *domV*, S2 loop, *ii*), the entire domain (S15 *domV*, *iii*), or stems a and b and connecting loops (S15a, b *domV*, *iv*) replaced by the corresponding region from S15 (1).

(**B, C**) Temperature sensitivity (B) and neurovirulence (C) of the mutant viruses were evaluated in Vero cells and by intraspinal (i.s.) inoculation of poliovirus into susceptible Tg66 mice, respectively (STAR methods).

Figure S2

A

	Cre1	Cre1a	Cre2	Cre3	Cre4	Cre5
ΔΔG:	0.0	-0.4	-2.8	-4.7	-6.9	-10.0

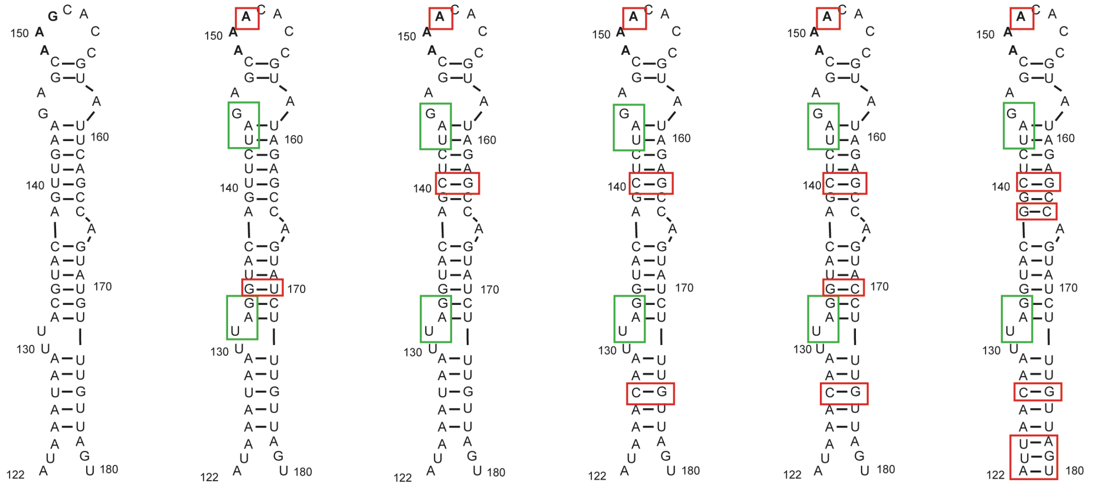

B

Temperature sensitivity

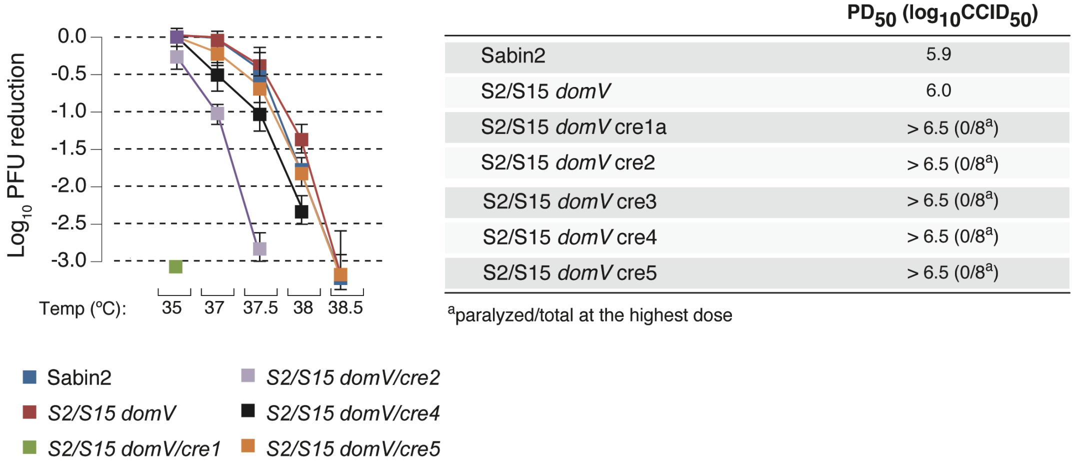

C

Neurovirulence

	Tg66-CBA, i.s.
	**PD_{50} ($log_{10}CCID_{50}$)**
Sabin2	5.9
S2/S15 *domV*	6.0
S2/S15 *domV* cre1a	> 6.5 (0/8[a])
S2/S15 *domV* cre2	> 6.5 (0/8[a])
S2/S15 *domV* cre3	> 6.5 (0/8[a])
S2/S15 *domV* cre4	> 6.5 (0/8[a])
S2/S15 *domV* cre5	> 6.5 (0/8[a])

[a]paralyzed/total at the highest dose

Figure S2. Related to Figure 1 and 5. CRE Optimization.

(**A**) RNA secondary structures of *cre* and serially optimized *cre* 1–5 with optimized base-pairs shown in red rectangles and "in-frame" STOP codons in green rectangles.

(**B, C**) Temperature sensitivity in Vero cells (B) and neurovirulence in Tg66 mice (C) of Sabin2 with *cre* mutants were compared to select sufficiently fit *cre5* for further analysis.

Figure S3

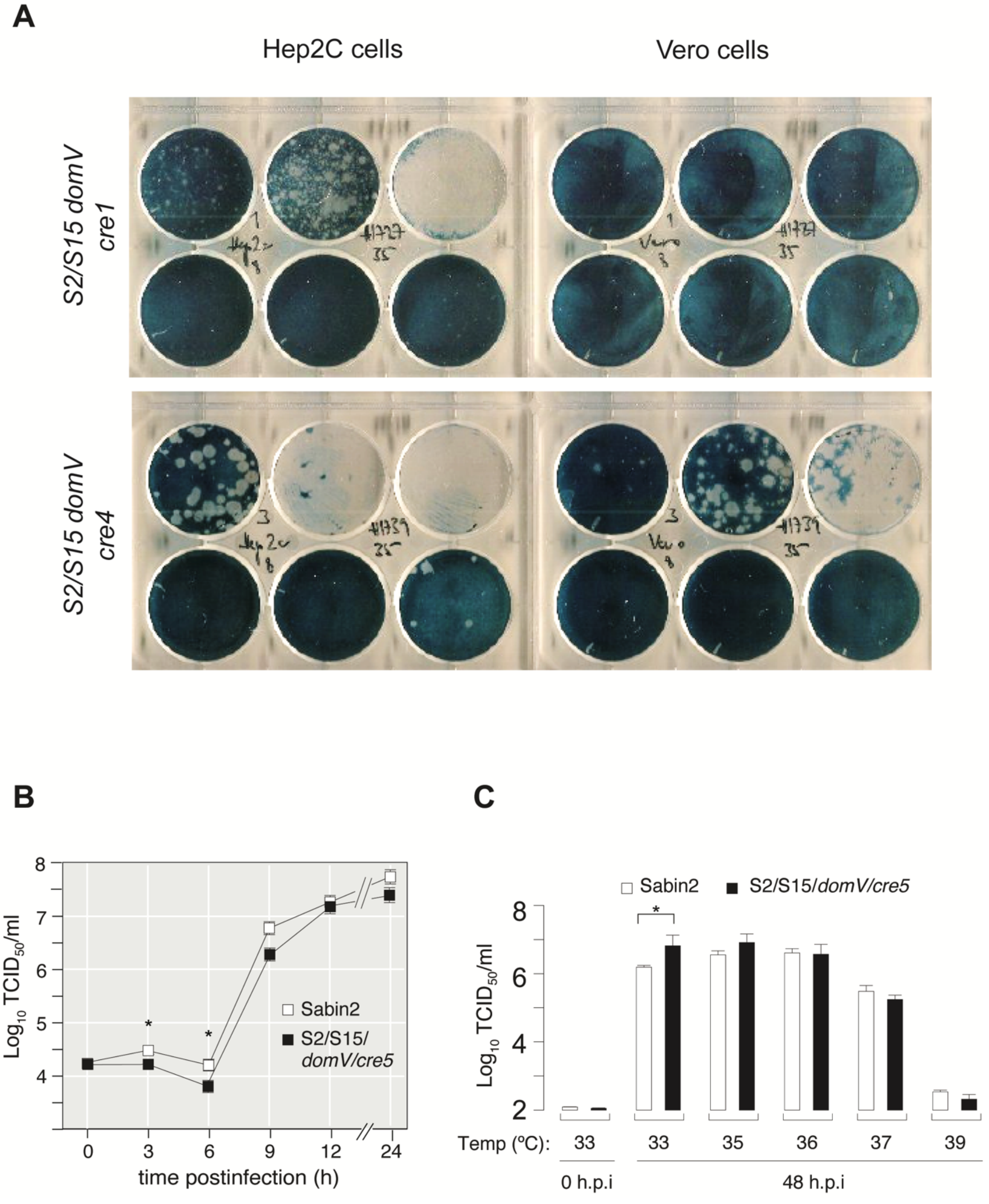

Figure S3. Related to Figure 1 and 5. Plaque Morphology and Replication Phenotype of *cre* Variants.

(**A**) Plaque morphology of S2/S15 *domV*/*cre1* and *cre4*. For each virus equivalent, 10-fold dilutions were plated from top-right to bottom-left in the 6-well plates of Hep2c and Vero cells. (**B**) Vero cells were infected with S2/S15 *domV*/*cre5* and Sabin2 viruses at m.o.i. of 10 at 33 ºC for one-step growth analysis. Asterisks indicate significant difference as compared to Sabin2; *p* values are 0.8796, 0.0238, 0.0136, 0.3419, 0.8796, and 0.5938 for 0, 3, 6, 9, 12, and 24-h post-infection, respectively.

(**C**) Vero cells were infected with S2/S15 *domV*/*cre5* and Sabin2 viruses at an m.o.i. of 0.01 under different temperatures and harvested 48 h after infection to compare virus replication yields. Asterisk represents significant difference as compared to Sabin2; $p = 0.0359$, 0.3061, 0.6433, 0.3165, and 0.4295 for 33, 35, 36, 37 and 39°C, respectively.

Figure S4

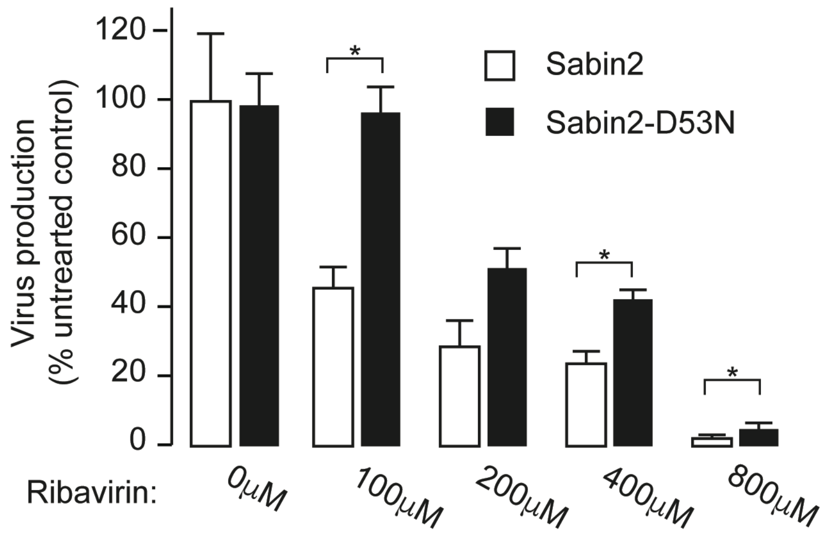

Figure S4. Related to Figure 2. Ribavirin Resistant Mutants.

To test for replication fidelity, we used a ribavirin-sensitivity test. Among the 11 fidelity candidates isolated, Sabin2-D53N showed the most significant increase in resistance to mutagen (ribavirin) of the tested concentrations. Asterisks indicate significant difference as compared to Sabin2; p = 0.9990, 0.0267, 0.1237, 0.0054, and 0.0065 for 0, 100, 200, 400, and 800 μM, respectively.

Figure S5

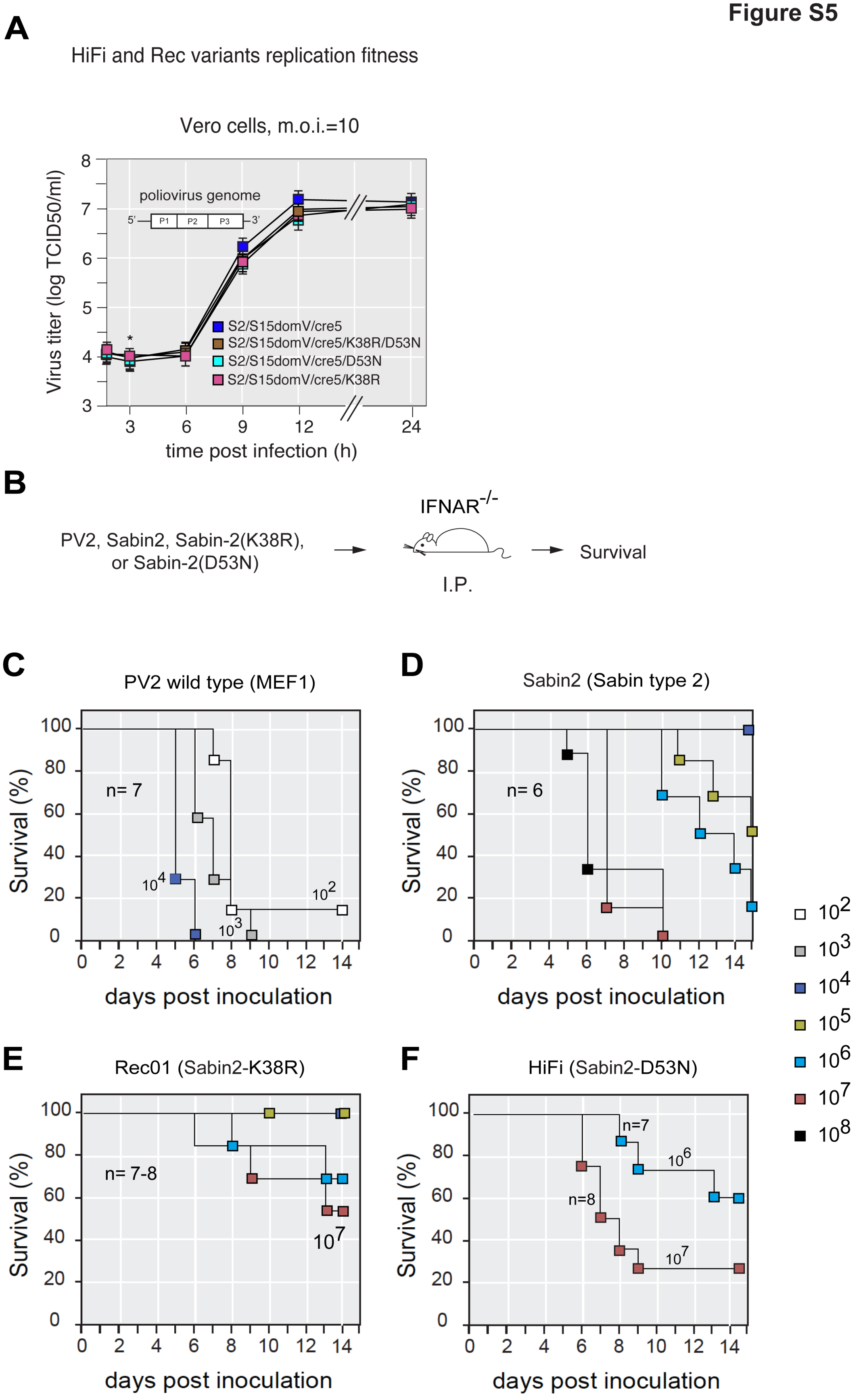
A
HiFi and Rec variants replication fitness
Vero cells, m.o.i.=10
poliovirus genome
5'
P1
P2
P3
3'
S2/S15domV/cre5
S2/S15domV/cre5/K38R/D53N
S2/S15domV/cre5/D53N
S2/S15domV/cre5/K38R
Virus titer (log TCID50/ml)
time post infection (h)
B
PV2, Sabin2, Sabin-2(K38R), or Sabin-2(D53N)
IFNAR-/-
I.P.
Survival
C
PV2 wild type (MEF1)
n= 7
D
Sabin2 (Sabin type 2)
n= 6
E
Rec01 (Sabin2-K38R)
n= 7-8
F
HiFi (Sabin2-D53N)
n=7
n=8
Survival (%)
days post inoculation
10^2
10^3
10^4
10^5
10^6
10^7
10^8

Figure S5. Related to Figure 2. Replication and Virulence of *HiFi* and *Rec1* Viruses.

(**A**) Effects of D53N and K38R mutation on Sabin2 replication were determined by one-step growth analysis by comparing replication kinetics. Vero cells were infected at an m.o.i. of 10 at 33 ºC. Line plots show means ± standard deviation (SD) of triplicates. * $p = 0.0017$ for difference between S2/S15doV/cre5 and S2/S15doV/cre5-K38R at 3-h post infection.

(**B**) Virulence of PV2, Sabin2 and Sabin2 mutants were compared in an IFNR-KO mouse model.

(**C-F**) Survival of mice intraperitoneally (i.p.) inoculated with PV2 wild type (C), Sabin2 (D), Sabin2-*Rec1* (E) and Sabin2-*HiFi3* (F) were monitored for 14 days. Log-rank test was performed to compare the virulence of Sabin2 and mutants. *p* values were 0.0076 for Rec1 and Sabin2 at doses of 10^7, 0.16 at 10^6, and 0.0387 at 10^5 (E). p value = 0.7757 for HiFi3 at doses of 10^7 and 0.3480 at 10^6 (F).

Figure S6

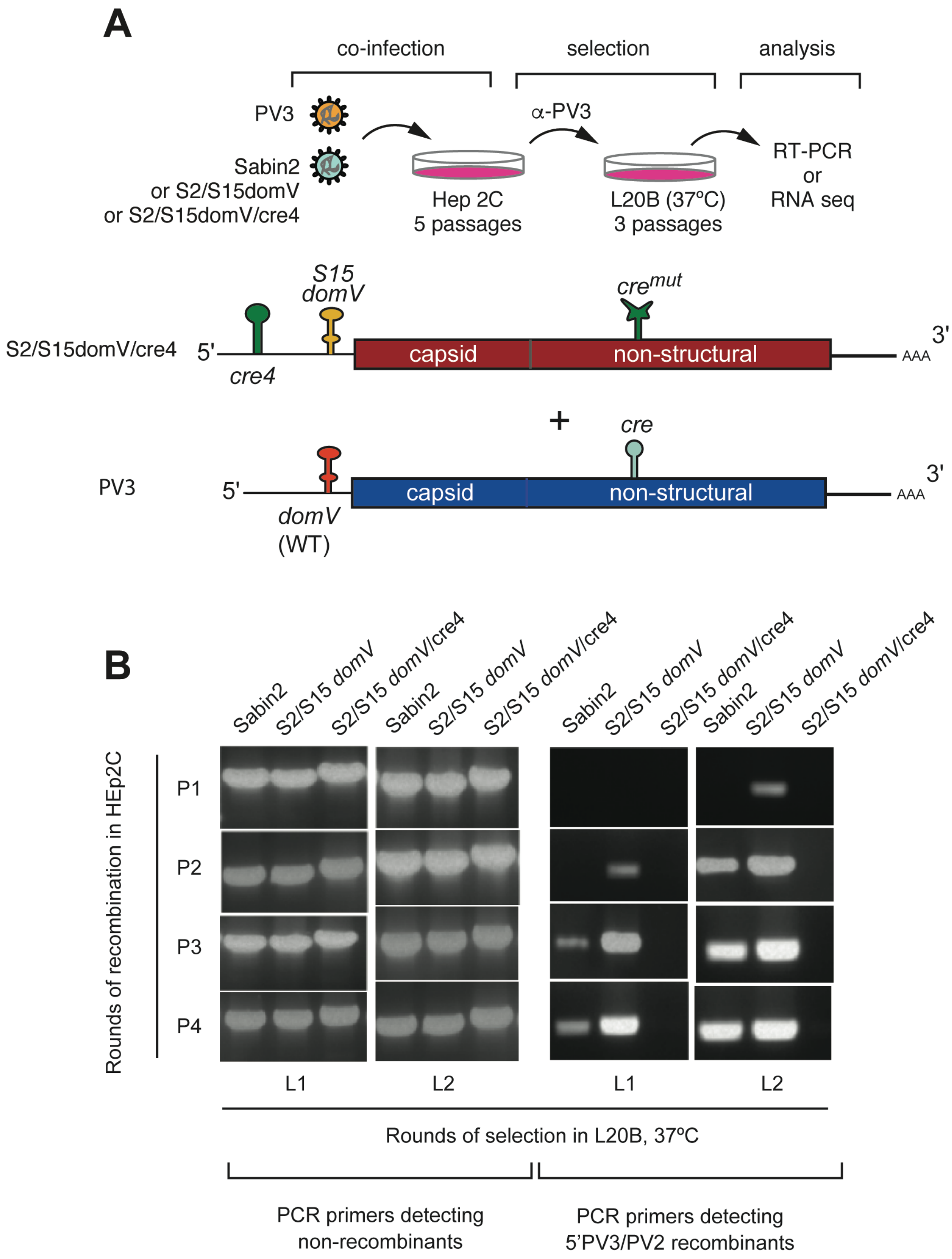

Figure S6. Related to Figure 5. Recombination Assay.

(**A**) Diagram of the experimental procedure to test the effects of CRE relocation on recombination. HEp2C cells were coinfected with PV3 plus one of the type 2 viruses at 33 ºC. Viral progeny was neutralized with anti-PV3 antibodies and further selected in L20B cells at 37 ºC after each HEp2c passage. The resulting virus populations were analyzed by RT-PCR and deep sequencing.

(**B**) Recombination between Sabin2 and PV3 was analyzed by RT-PCR after each passage in Hep2C (P1-P4) and after selection in L20B cells (L1-L2) or full-length NGS. Primer sets specific to non-recombinants (left panel), and 5′PV3/PV2 recombinants (right panel) were used in RT-PCR analysis.

Figure S7

	composition of domV	PD50 (log $CCID_{50}$)
PV3 + Sabin2	40% PV3; 60% Sabin-2	1.8
PV3 + S2/S15domV	70% PV3; 30% S2/S15domV	1.8
PV3 + S2/S15domV/cre5	100% S2/S15domV/cre5	5.6

Figure S7. Related to Figure 5. Characterization of Progeny Virus from Recombination Assay.

Sequence composition of *domV* in virus populations obtained after five recombination passages in HEp2c cells and two selection passages in L20B cells (H5L2) were determined by deep sequencing. Percentage of *domV* sequence mapped to PV3, Sabin2, S2/S15*domV*, or S2/S15*domV/cre5* is shown. Levels of neurovirulence of the obtained virus populations are compared between conditions using PD_{50} determined with the Tg66 mouse model.
